# The WID-BC-index identifies women with primary poor prognostic breast cancer based on DNA methylation in cervical samples

**DOI:** 10.1038/s41467-021-27918-w

**Published:** 2022-02-01

**Authors:** James E. Barrett, Chiara Herzog, Allison Jones, Olivia C. Leavy, Iona Evans, Susanne Knapp, Daniel Reisel, Tatiana Nazarenko, Yoo-Na Kim, Dorella Franchi, Andy Ryan, Joanna Franks, Line Bjørge, Michal Zikan, David Cibula, Nadia Harbeck, Nicoletta Colombo, Frank Dudbridge, Louise Jones, Karin Sundström, Joakim Dillner, Angelique Flöter Rådestad, Kristina Gemzell-Danielsson, Nora Pashayan, Martin Widschwendter

**Affiliations:** 1 European Translational Oncology Prevention and Screening (EUTOPS) Institute, Universität Innsbruck, Innsbruck, Austria, Hall in Tirol Austria; 2 Department of Women’s Cancer, University College London, London, UK; 3 Institute for Biomedical Aging Research, Universität Innsbruck, Innsbruck, Austria; 4 Department of Health Sciences, University of Leicester, Leicester, LE1 7RH UK; 5 Department of Non-communicable Disease Epidemiology, London School of Hygiene and Tropical Medicine, London, UK; 6 Istituto Europeo di Oncologia, IRCCS, Milan, Italy; 7 Breast Service, University College London Hospital, London, UK; 8 Department of Obstetrics and Gynaecology, Haukeland University Hospital, Bergen, Norway; 9 Centre for Cancer Biomarkers CCBIO, Department of Clinical Science, University of Bergen, Bergen, Norway; 10 Department of Gynecology and Obstetrics, First Faculty of Medicine, Charles University in Prague, University Hospital Bulovka, Prague, Czech Republic; 11 Gynaecologic Oncology Center, Department of Obstetrics and Gynecology, First Faculty of Medicine, Charles University in Prague, General University Hospital in Prague, Prague, Czech Republic; 12 Breast Center, Department of Obstetrics and Gynecology, University of Munich (LMU), Munich, Germany; 13 University of Milano-Bicocca, Milan, Italy; 14 Centre for Tumour Biology Department, Barts Cancer Institute, Queen Mary University of London, London, UK; 15 Department of Laboratory Medicine, Division of Pathology, Karolinska Institutet, Stockholm, Sweden; 16 Department of Women’s and Children’s Health, Karolinska Institutet and Karolinska University Hospital, Stockholm, Sweden; 17 Department of Applied Health Research, University College London, London, UK

**Keywords:** Breast cancer, Translational research

## Abstract

Genetic and non-genetic factors contribute to breast cancer development. An epigenome-based signature capturing these components in easily accessible samples could identify women at risk. Here, we analyse the DNA methylome in 2,818 cervical, 357 and 227 matched buccal and blood samples respectively, and 42 breast tissue samples from women with and without breast cancer. Utilising cervical liquid-based cytology samples, we develop the DNA methylation-based Women’s risk IDentification for Breast Cancer index (WID-BC-index) that identifies women with breast cancer with an AUROC (Area Under the Receiver Operator Characteristic) of 0.84 (95% CI: 0.80–0.88) and 0.81 (95% CI: 0.76–0.86) in internal and external validation sets, respectively. CpGs at progesterone receptor binding sites hypomethylated in normal breast tissue of women with breast cancer or in *BRCA* mutation carriers are also hypomethylated in cervical samples of women with poor prognostic breast cancer. Our data indicate that a systemic epigenetic programming defect is highly prevalent in women who develop breast cancer. Further studies validating the WID-BC-index may enable clinical implementation for monitoring breast cancer risk.

## Abstract (web summary)

Breast cancer is most commonly diagnosed via a needle biopsy. In this study, the authors show that cervical samples from women with breast cancer have a methylation signature different to that of healthy controls.

## Introduction

Breast cancer is by far the most common cancer in females in general, and a leading cause of death in young women^1^. To date, the identification of individuals with primary cancer is achieved by assessing evidence directly from the tumour (e.g. imaging^2^ or detection of cancer cell products released into the system^3,4^). Currently, available early detection strategies, such as mammography screening, suffer from low performance in young women, over-diagnosis, and decreasing attendance rates, and its benefit on mortality reduction has recently been questioned^5^. High-risk prevention strategies and risk-stratified screening require high discriminatory accuracy, that can only be achieved with predictors much stronger than those yet discovered^6^. Yet the best predictive models combining non-genetic factors and a polygenic risk score have only led to a Receiver Operator Characteristic (ROC) Area Under the Curve (AUC) of 0.68^7^. In contrast, cervical cancer screening (i.e. assessing cervical samples) that offers simple non-invasive access to precancerous cells has reduced the incidence and mortality from cervical cancer by more than 50%^8^.

Epigenetic (i.e. DNAme) changes have been identified in normal breast tissue adjacent to breast cancers^9^ and could potentially serve as a surrogate for both genetic and non-genetic factors including lifestyle, reproductive and environmental exposures contributing to breast cancer development^10^. A number of proof of principle studies, so far exclusively performed in blood, have demonstrated that certain DNAme changes are associated with breast cancer predisposition^11–16^. Steroid hormones are essential drivers of breast cancer formation and hence it is a requirement that surrogate tissue is sufficiently hormone sensitive. For instance, prolonged^17^ or higher^18^ exposure to progesterone is strongly associated with the formation of poor prognostic breast cancer^17–22^ and the progesterone receptor antagonist mifepristone effectively prevents breast cancer formation in mice^23^.

Sample heterogeneity and the choice of surrogate tissue are deemed to be among the most important factors for impending clinical implementation^24^. Thus, we aimed to assess whether DNAme profiles derived from cervical liquid-based cytology samples (i.e. containing hormone-sensitive epithelial cells, which are capable of recording breast cancer-predisposing factors at the level of the epigenome^24^ and can be self-collected), are able to identify women with primary breast cancer.

Here, we perform an epigenome-wide DNAme analysis in cervical liquid-based cytology samples from women who had recently been diagnosed with breast cancer and in matched controls. We establish the WID-BC test (Women’s risk IDentification for Breast Cancer index) and validate it in independent sets of cervical samples. We also carry out further validation in samples from women with ovarian cancer and a set of matched cervical, buccal, and blood samples from *BRCA* mutation carriers and in archived samples from a population cohort. In addition, we specifically assess DNAme at progesterone receptor binding sites (PR-BS) in breast and cervical samples to evaluate whether systemic factors driving carcinogenesis in the breast can be captured by assessment of the cervical epigenome.

## Results

### Sample heterogeneity and differential methylation

**Fig. 1:**
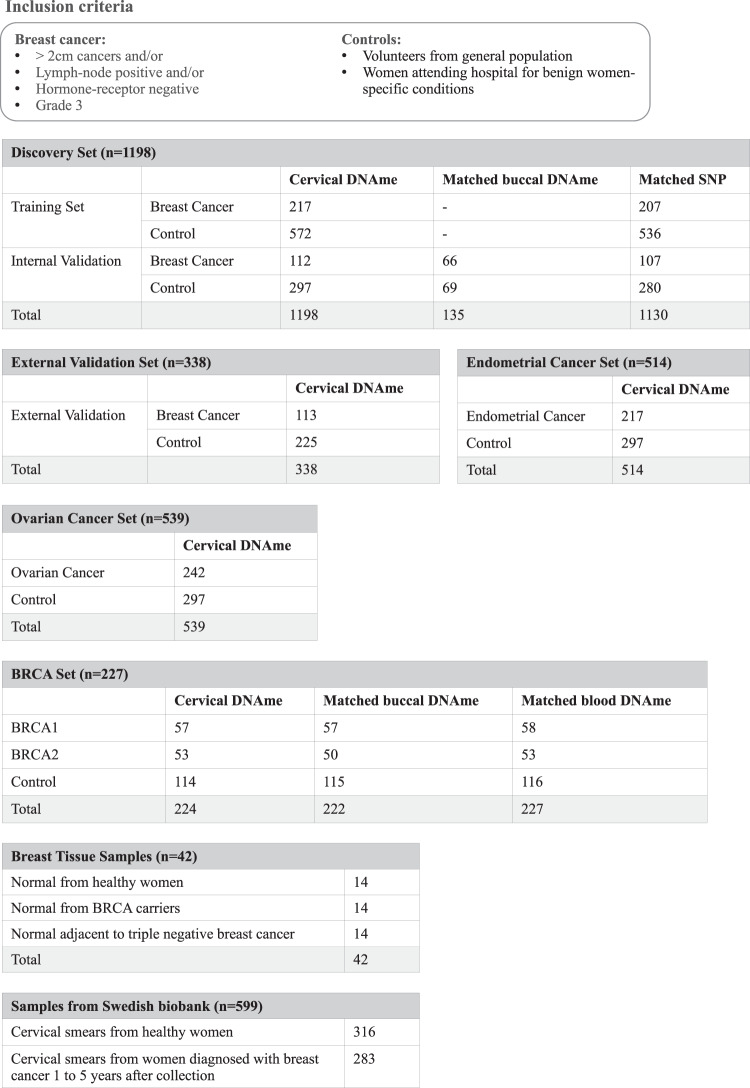
Overview of experimental design and datasets. We included women with breast cancer who had at least one poor prognostic feature and recruited both hospital-based controls and controls from the general population. The discovery set was further subdivided into a training set and internal validation set and was used to derive the WID-BC-index. For a subset of individuals in the internal validation set, matched buccal samples and SNP data were available. The WID-BC-index was evaluated in the external validation set. Additional datasets for evaluation of the WID-BC-index consisted of cervical samples from individuals with endometrial or ovarian cancer, or *BRCA* mutation carriers, as well as breast tissue samples from healthy controls, *BRCA* mutation carriers, or normal tissue adjacent to triple-negative breast cancer. Predictive assessment of the WID-BC-index was carried out using samples from a Swedish Biobank which included cervical smears from healthy controls and women who were diagnosed with breast cancer 1 to 5 years after sample collection. Source data are provided as a Source Data file.

For the discovery set, we collected samples from 329 women with primary breast cancer with poor prognosis features (defined by >2 cm cancers and/or lymph-node positive and/or hormone-receptor negative and/or grade 3) from 14 European centres at the time of diagnosis and before treatment commenced, and 869 women without breast cancer (593 from the general population and 276 from women attending hospital for benign women-specific conditions) (Fig. 1). Epidemiological and clinical characteristics in the discovery set are presented in Supplementary Tables 1 and 3. Epigenome-wide DNAme was analysed using an Illumina Infinium EPIC bead chip array which encompasses over 850,000 CpG sites^25^.

**Fig. 2:**
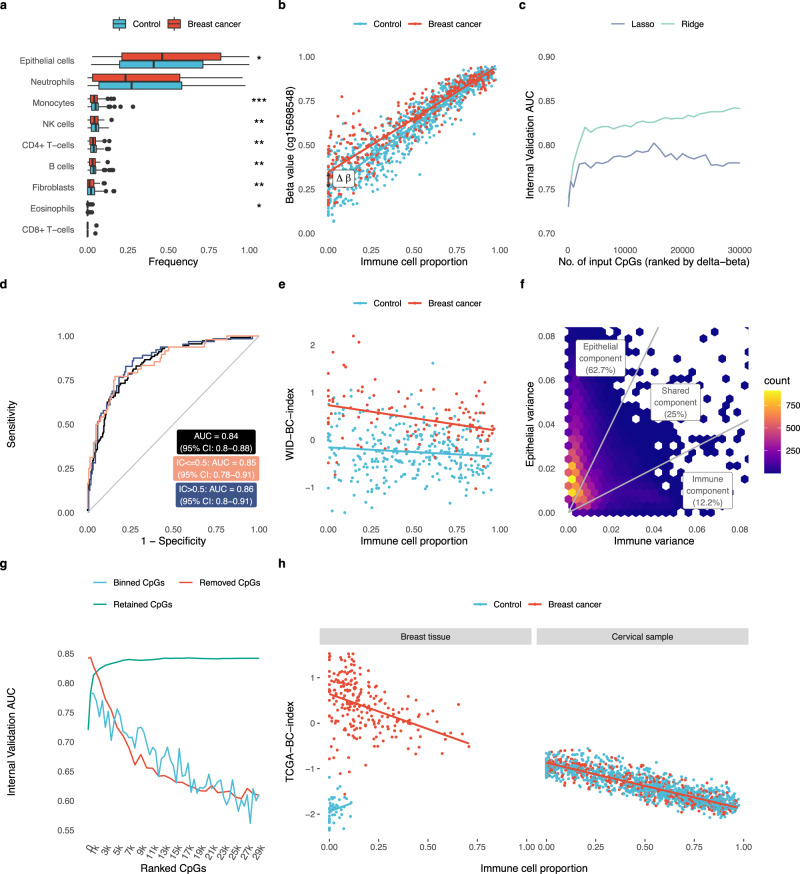
Development of an index discriminating breast cancer cases from controls based on cervical samples. **a** Distribution of different cell types in samples of the discovery dataset inferred using the HEpiDISH algorithm (*p < 0.05, **p < 0.01, ***p < 0.001 in two-sided Wilcoxon signed-ranked test; *n* = 869 controls, 329 breast cancer cases). Exact *p* values: epithelial cells, *p* = 0.030; neutrophils, *p* = 0.052; monocytes, *p* = 0.0009; NK cells, 0.006; CD4+ T cells, *p* = 0.0013; B cells, *p* = 0.002; fibroblasts, *p* = 0.001; eosinophils, 0.029; CD8+ T cells, 0.475. No adjustment for multiple comparisons was made. Box plots correspond to standard Tukey representation, with boxes indicating mean and interquartile ranges, and lines indicating smallest and largest values within 1.5 times of the 25^th^ and 75^th^ percentile, respectively. Dots indicate outlier values. **b** Example of a CpG with cell-type-specific methylation. **c** Area under the receiver operating characteristic curve (AUROC) in the internal validation set as a function of the number of CpGs used to train the classifier. **d** ROC curves of the WID-BC-index in the internal validation set for samples with an immune cell proportion ≤0.5 and >0.5. **e** Distribution of the WID-BC-index with respect to immune cell proportion in the internal validation set. **f** Distribution of the estimated variance in epithelial and immune cells across all CpGs used in the WID-BC-index. **g** AUC values in the internal validation set after training classifiers on different subsets of the CpGs used in the WID-BC-index. The top n CpGs were either retained or removed. CpGs were also split into separate bins of size 500. **h** An index developed using data extracted from TCGA breast cancer and normal samples (TCGA-BC-index) is able to discriminate cancer cases from controls in breast tissue (*n =*44 controls, *n* = 222 breast cancer samples) but not cervical samples (*n* = 1094 controls, *n* = 442 breast cancer cases), prompting the development of a cervix-specific index reflecting systemic changes in a hormone-sensitive surrogate tissues. Source data are provided as a Source Data file.

We assessed the level of cell-type heterogeneity in each cervical sample using EpiDISH^26^, an algorithm that infers the relative proportion of epithelial cells, fibroblasts, and seven subtypes of immune cells in each sample. Immune cell proportion followed an approximately uniform distribution in both cancer cases and controls. There was a significantly greater proportion of epithelial cells in cancers, and correspondingly fewer immune cells across all subtypes (specifically pronounced for monocytes) in the discovery dataset (Fig. 2a). This difference was comparatively small, however, and absent in the external validation dataset (Supplementary Fig. 1).

Identifying CpGs with differential methylation between cases and controls was hampered by sample heterogeneity, since any differential methylation specific to epithelial cells was greatly diminished in samples with high immune cell proportion (see example in Fig. 2b). We therefore used a linear model to estimate epithelial- and immune-specific differentially methylated CpGs.

### Development of discriminatory index

To derive a signature discriminating between women with breast cancer and those without based on  cervical methylation, the WID-BC-index, we used ridge and lasso regression to classify individuals as cases or controls. Classifiers were trained on two-thirds of the discovery dataset (572 cancer-free controls, 217 breast cancer cases) and the remaining one-third was used as an internal validation set (297 controls, 112 cases) (Fig. 1). The internal validation set was used to determine the optimal number of CpGs used to construct the index. The AUC was used as a measure of predictive performance. Once the classifier was finalised a completely independent external validation set was used to evaluate its performance.

Predictive performance was evaluated as a function of *n*, the number of CpGs used to train the classifier, using the internal validation dataset (Fig. 2c) and optimal performance of 0.84 (95% CI: 0.80–0.88) was achieved using 29,000 CpGs with ridge regression (Fig. 2d). The WID-BC-index was moderately, but significantly associated with immune cell proportion fraction in the internal validation set (Fig. 2e, linear regression coefficients of −0.55, *p* = 0.004 and −0.20, *p* = 0.07 in cases and controls, respectively).

In samples with an immune cell proportion fraction ≤0.5 and immune cell proportion >0.5 the AUC was 0.85 (95% CI: 0.78–0.91) and 0.86 (95% CI: 0.80–0.91), respectively, suggesting that discriminatory signals are present in both epithelial and immune cells. We used a statistical model to estimate epithelial- and immune-specific variability at each of the 29,000 CpGs and divided them into “epithelial” (18,190 CpGs), “immune” (3,533 CpGs) or “shared” (7,277 CpGs) subsets as shown in Fig. 2f.

Since the WID-BC-index is defined as a weighted sum of 29,000 beta-values the index can be split into three subcomponents by taking the weighted sums corresponding to each of the three subsets of CpGs (Supplementary Fig. 2a). The epithelial subcomponent captures an epithelial-specific signal in the internal validation set, the immune subcomponent corresponds to an immune-specific signal, whereas the shared subcomponent captures a signal that is shared across both cell types (Supplementary Fig. 2b–d). We evaluated the AUC in the internal validation set after omitting each subcomponent and observed that the removal of the epithelial component leads to the greatest reduction in performance suggesting that this component is particularly informative (Supplementary Fig. 2e). We found that the index was highly depleted of CpG islands and enriched for open sea regions (Supplementary Fig. 2f).

In addition, we ranked the 29,000 CpGs used to define the WID-BC-index according to the absolute value of the regression coefficients from the ridge model. In order to assess how informative the top CpG sites are we trained sub-classifiers on the top *n* sites (Fig. 2g). We observed that AUCs of 0.78 and 0.81 can be achieved with the top 500 and 1000 CpGs, respectively, indicating that the top-ranked subsets are particularly informative. We also trained sub-classifiers after removing the top *n* CpGs, and on subsets of 500 CpGs after partitioning the ranked list into bins of size 500. In both cases, we found that a substantial predictive signal is present.

### Performance of a breast cancer tissue signature in cervical samples

In order to assess whether any breast cancer tissue-specific changes (e.g. via cell-free DNA) can be detected in the cervix, we developed a breast cancer-specific epigenetic signature based on data from the TCGA-BRCA programme by applying lasso regression on the top differentially methylated CpGs between normal tissue and primary tumour tissue. This signature, the TCGA-Breast Cancer index (TCGA-BC-index), consisted of 31 CpGs and was able to discriminate non-cancerous from breast cancer tissue perfectly in a validation set, but did not discriminate controls from breast cancer cases in cervical samples (Fig. 2h). This indicated that the observed WID-BC index does not appear to be driven by epigenetic changes which are present in the actual breast cancer itself.

### Validation in independent datasets

**Fig. 3:**
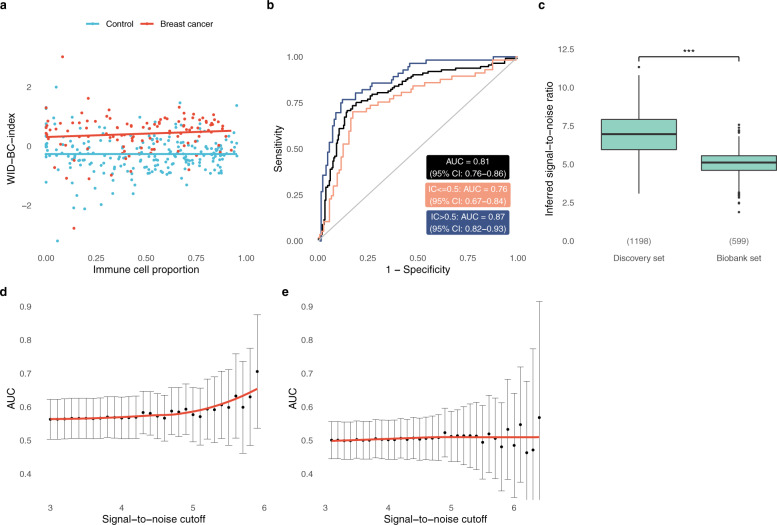
The WID-BC-index discriminates poor-prognostic breast cancer cases from controls in cervical samples but is negatively impacted by sample degradation in biobanked samples. **a** The WID-BC-index versus immune cell proportion in the external validation set. **b** ROC curve in the external validation set (*n* = 225 controls, *n* = 113 breast cancer cases). **c** Inferred signal-to-noise ratio in the discovery and biobank datasets. Numbers in brackets indicate sample numbers (*n* = 1198 in discovery set, 599 in biobank set). ***p < 2.2e-16 in two-sided Wilcoxon signed-rank test. Box plots correspond to standard Tukey representation, with boxes indicating mean and interquartile range, and lines indicating smallest and largest values within 1.5 times of the 25^th^ and 75^th^ percentile, respectively. Dots indicate outlier values. **d** The AUC when discriminating between healthy controls and poor-prognostic breast cancer samples from the Swedish cytology biobank dataset as a function of signal-to-noise cutoff. *n* = 316 controls, 131 poor prognostic breast cancer cases. **e** AUCs corresponding to non-poor prognostic samples from the biobank dataset. *n* = 316 controls, *n* = 152 non-poor prognostic breast cancer cases. Error bars in **d**, **e** indicate upper and lower 95% confidence intervals, respectively. Source data are provided as a Source Data file.

A separate independent external validation dataset consisting of 225 controls and 113 breast cancer cases was used to validate the index performance (Fig. 1, epidemiological and clinical characteristics in Supplementary Tables 2 and 3). The WID-BC-index was computed for each woman (Fig. 3a) resulting in an AUC of 0.81 (Fig. 3b; 95% CI: 0.76–0.86). There was no significant dependence on immune cell proportion.

We next analysed cervical samples from yet unaffected *BRCA1* (*n* = 57) and *BRCA2* (*n* = 53) mutation carriers (i.e. women who have not yet developed breast and/or ovarian cancer) and 114 healthy controls. In mutation carriers of *BRCA1* and *BRCA2* (whose breast cancer risk is slightly lower and starts to increase later than that of *BRCA1* carriers^27^) we observed AUCs of 0.61 (95% CI: 0.52–0.69) and 0.55 (95% CI: 0.46–0.64), respectively (Supplementary Fig. 3). The inferred cellular composition of these samples was comparable to the external validation dataset (Supplementary Fig. 1b–e).

To study the predictive performance of WID-BC-index, we analysed Karolinska University Laboratory cervical cytology biobank samples from women who subsequently developed breast cancer a median of 351 days (range 1–1704 days) after the cervical sample collection. To assess the impact of the storage protocol on DNAme (storage time in methanol-containing PreservCyt at −25  °C ranged from 1,252 to 2,877 days), we developed a statistical technique to infer the signal-to-noise ratio of each sample (Supplementary Methods). The biobanked samples suffered from a lower signal-to-noise ratio compared to the discovery set (Fig. 3c).

Despite this degradation, in 131 poor prognostic breast cancer cases (defined as >2 cm tumours and lymph node positive; hormone receptor status and grade were unknown) we estimated an AUC > 0.7 in samples with the highest signal-to-noise levels (Fig. 3d; Supplementary Fig. 2g). In 154 cases not classified as poor prognostic we did not detect any discriminatory signal (Fig. 3e; Supplementary Fig. 2h).

These findings suggest that the biobanked samples suffered substantial degradation and that the discriminatory signal may have been masked by background noise in the lowest quality samples. We conclude that it is not possible to fully validate the WID-BC-index in currently available cohort-based long-term archived samples stored in methanol-based fluid at >−30 °C.

### Performance of index in matched buccal and blood samples

DNAme is highly tissue-specific and specific exposures are recorded in certain cell subtypes^24,28,29^. The index decomposition in Fig. 2f implies that the WID-BC-index uses epithelial- and immune-specific signals as well as a shared component. In order to assess whether the WID-BC-index (derived from cervical liquid-based cytology samples) can be extended to other tissue types we analysed matched buccal and blood samples from the *BRCA* dataset above (Fig. 1). Similar to the cervical samples, a substantial proportion of buccal DNA originates from immune cells (Supplementary Fig. 1). In *BRCA1* carriers we found an AUC of 0.67 (95% CI: 0.59–0.76) in buccal samples, and 0.64 (95% CI: 0.56–0.72) in blood (Supplementary Fig. 3). In *BRCA2* carriers the AUC was 0.61 (95% CI: 0.52–0.71) in buccal samples and 0.60 (95% CI: 0.51–0.69) in the blood (Supplementary Fig. 3).

**Fig. 4:**
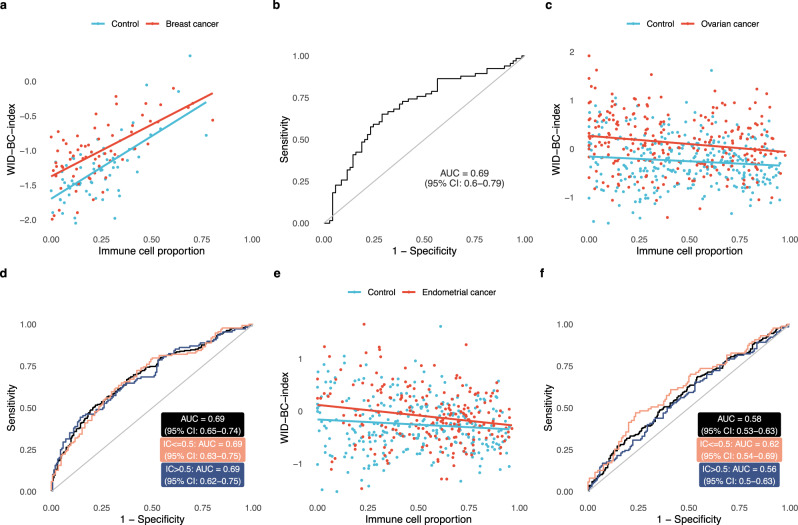
WID-BC-index performance in buccal samples and in other gynaecological cancer patients. **a** WID-BC-index versus immune cell proportion in matched buccal samples from the breast cancer internal validation set (*n* = 69 controls, *n* = 66 breast cancer cases). **b** ROC curve corresponding to matched buccal samples (*n* = 69 controls, *n* = 66 breast cancer cases). **c** WID-BC-index versus immune cell proportion in ovarian cancer cases and healthy controls (*n* = 297 controls, *n* = 242 ovarian cancer cases). **d** ROC curve corresponding to the ovarian cancer samples (*n* = 297 controls, *n* = 242 ovarian cancer cases). **e** WID-BC-index versus immune cell proportion in endometrial cancer cases (*n* = 297 controls, *n* = 217 endometrial cancer cases) and **f** the corresponding ROC curve (*n* = 297 controls, *n* = 217 endometrial cancer cases). Source data are provided as a Source Data file.

We additionally analysed matched buccal samples from a subset of 135 women in the internal validation set (69 controls and 66 cases). We found that the discriminatory signal derived using cervical samples was also present in these matched buccal samples (Fig. 4a), yielding an AUC of 0.69 (Fig. 4b; 95% CI: 0.60–0.79). There was a correlation of 0.57 (*p* < 10^−12^) between the WID-BC-index computed in matched cervical and buccal samples. The immune and shared subcomponents showed the greatest discriminatory performance (Supplementary Fig. 4a–c). We did not observe any differences in inferred cellular composition between cancer cases and controls in these matched buccal samples (Supplementary Fig. 1f).

### Validation in women with ovarian or endometrial cancer

Women with breast cancer are also at higher risk for ovarian and endometrial cancer^30^: the risk to develop either ovarian and endometrial cancer is higher in women who develop breast cancer at an early and later age, respectively^30^.

Hence, we additionally analysed 242 and 217 cervical samples from women with ovarian and endometrial cancer, respectively (Fig. 1). The clinical and epidemiological characteristics of these datasets are presented in Supplementary Tables 4–7. Of note, these samples displayed a slight but significant difference in inferred cellular composition compared to controls (Supplementary Fig. 1g, h). We obtained an AUC of 0.69 (95% CI: 0.65–0.74; Figs. 4c and 3d) and 0.58 (95% CI: 0.53-0.63; Fig. 4e, f) in these ovarian and endometrial cancer cases, respectively, and the same 297 control samples from the breast cancer internal validation set. The subcomponents for the ovarian cancer set followed a similar pattern as the breast cancer cases (Supplementary Fig. 4d, e).

For each of the validation datasets we computed odds ratios corresponding to quartiles of the WID-BC-index (Table 1).

**Table 1:** Odds ratios corresponding to quartiles defined using the internal validation breast cancer dataset.

Quantile	Control	Cancer	OR (unadjusted)	OR (adjusted)
**Breast cancer internal validation**				
(−1.53, −0.58)	75	2	1.00 (reference)	1.00 (reference)
(−0.58, −0.28)	74	5	2.42 (0.48,19.25)	2.29 (0.45, 17.15)
(−0.28, 0.07)	74	17	8.01 (2.17,56.31)	8.47 (2.23, 55.81)
(0.07, 1.62)	74	88	41.11 (12.33,274.77)	41.73 (12.2, 262.62)
**Breast cancer external validation**				
(−1.53, −0.58)	58	8	1.00 (reference)	1.00 (reference)
(−0.58, −0.28)	69	8	0.84 (0.29,2.46)	0.89 (0.3, 2.67)
(−0.28, 0.07)	50	14	2.00 (0.78,5.46)	2.57 (0.95, 7.51)
(0.07, 1.62)	48	83	12.19 (5.62,29.86)	15.67 (6.59, 42.38)
**Ovarian cancer**				
(−1.53, −0.58)	75	30	1.00 (reference)	1.00 (reference)
(−0.58, −0.28)	74	31	1.05 (0.57,1.91)	0.93 (0.48, 1.79)
(−0.28, 0.07)	74	51	1.72 (0.99,3.01)	1.16 (0.61, 2.19)
(0.07, 1.62)	74	130	4.36 (2.64,7.36)	3.27 (1.9, 5.68)
**Endometrial cancer**				
(−1.53, −0.58)	75	39	1.00 (reference)	1.00 (reference)
(−0.58, −0.28)	74	42	1.09 (0.63,1.88)	0.89 (0.46, 1.72)
(−0.28, 0.07)	74	61	1.58 (0.95,2.66)	0.74 (0.36, 1.49)
(0.07, 1.62)	74	75	1.94 (1.18,3.23)	0.80 (0.41, 1.53)

For the breast cancer datasets adjustment was based on a logistic regression model with age, menopausal status, age at menarche, number of first-degree relatives with breast cancer, and BMI included as covariates. For the ovarian and endometrial cancer datasets age and menopause were included as covariates. Odds ratios were estimated using median-unbiased estimation. Source data are provided as a Source Data file.

### Association with epidemiological and clinical factors

**Fig. 5:**
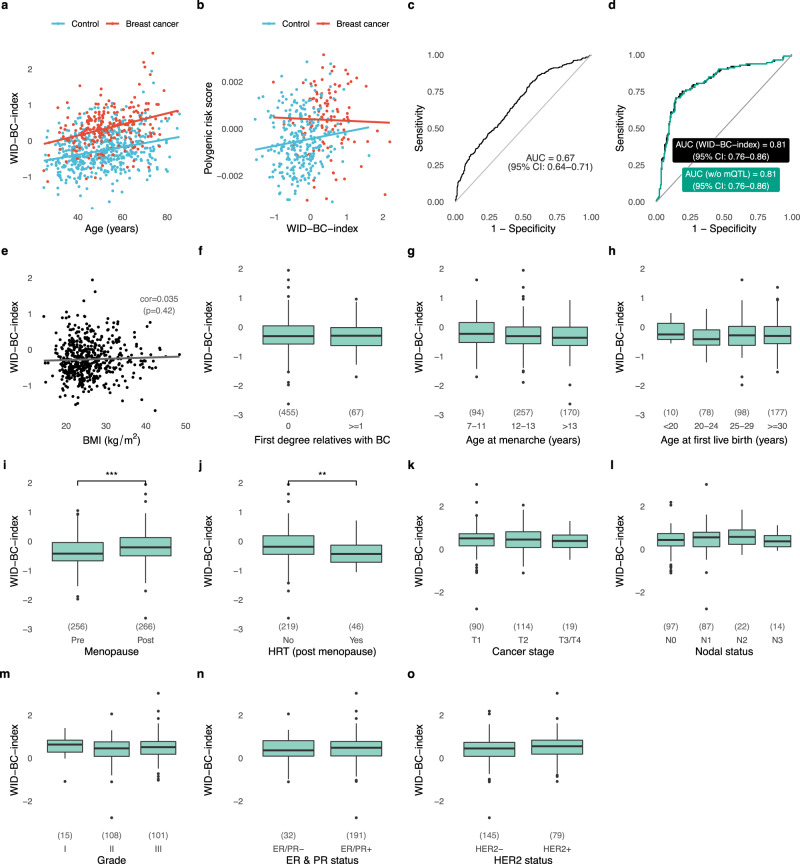
Association of the WID-BC-index with clinical and epidemiological parameters. **a** The WID-BC-index versus age in samples from the internal and external validation datasets. **b** Correlation with a polygenic risk score (PRS) based on 303 SNPs in the internal validation dataset. **c** ROC curve corresponding to the PRS evaluated in a subset of samples from the discovery set. **d** ROC curves of the WID-BC-index (black) or the WID-BC-index without 78 mQTLs previously associated with breast cancer (turquoise). Removal of mQTLs did not significantly influence performance of the WID-BC-index. **e** Correlation with body mass index (BMI) in controls. Assessment of the WID-BC-index with **f** family history, **g** age at menarche, **h** age at first live birth, **i** menopause, **j** hormone replacement therapy, **k** stage, **l** nodal status, **m** grade, **n** ER/PR status, and **o** HER2 status. **e**–**j** are based on controls from the internal and external validation datasets. ***p = 1.937e-05 in two-sided Welch’s *t*-test (Menopausal status), **p = 0.001 in two-sided Welch’s *t*-test (HRT). Box plots throughout (**f**–**o**) correspond to standard Tukey representation, with boxes indicating mean and interquartile range, and lines indicating smallest and largest values within 1.5 times of the 25^th^ and 75^th^ percentile, respectively. Dots indicate outlier values. No corrections for multiple adjustment were carried out. Source data are provided as a Source Data file.

We investigated the relationship between the WID-BC-index and various epidemiological variables using the internal and external validation datasets (the training dataset was not suitable because it was used to develop the index). A statistically significant association was found between the WID-BC-index and age (Fig. 5a, correlation coefficients of 0.27, *p* < 10^−9^ and 0.33, *p* < 10^−6^ in controls and cases respectively). The Illumina 650k Infinium Global Screening Array was used to genotype matched blood samples from a subset of 314 cases and 816 controls in the discovery set. We computed a recently published polygenic risk score (PRS; 303 of the 313 SNPs described^31^ were used) for breast cancer prediction. In the 107 cases and 280 controls from the internal validation set we found a modest but significant correlation of 0.13 (*p* = 0.03) between the PRS and the WID-BC-index (Fig. 5b) in controls and no significant correlation in cases (correlation coefficient −0.03, *p* = 0.7). In all samples of the discovery set for which PRS data were available  the AUC was 0.67 (Fig. 5c; 95% CI: 0.64–0.71). We also assessed whether removal of recently described methylation quantitative loci (mQTLs) associated with breast cancer risk influenced the index. Ho et al.^32^ assessed 235 of the 313 breast cancer variants^31^ with minor allele frequencies higher than 5% and based on this identified 822 cis-mQTLs of which 704 were present in our analysis set. Interestingly, 78 of these mQTLs were included in the WID-BC-index, which is more than expected by chance (observed to expected ratio 2.97 [95%CI 2.32–3.76, *p* < 0.001]); however removal of these 78 mQTLs from the WID-BC-index did not result in a significant drop in performance (Fig. 5d). In control samples no significant association was found between the WID-BC-index and BMI (Fig. 5e), individuals with 0 and ≥1 first-degree-relatives with breast cancer (Fig. 5f), age of menarche (Fig. 5g), or age at first live birth (Fig. 5h). The index was significantly higher in postmenopausal women (Fig. 5i, *p* < 10^−5^) and women who had not undergone hormone replacement therapy (Fig. 5j, p < 0.001). In breast cancer cases there was no association between the WID-BC-index and clinico-pathological features of the cancers (Fig. 5m–o).

No significant association was found between the WID-BC-index and various technical parameters including the time between sample collection and processing (Supplementary Fig. 5a), date of processing, plate number (samples were processed on 96 sample plates) and sentrix position. No difference was found between control samples from healthy volunteers and women presenting at hospitals for benign women-specific conditions (Supplementary Fig. 5b).

### The WID-BC-index is reflective of a fat-cell differentiation

**Fig. 6:**
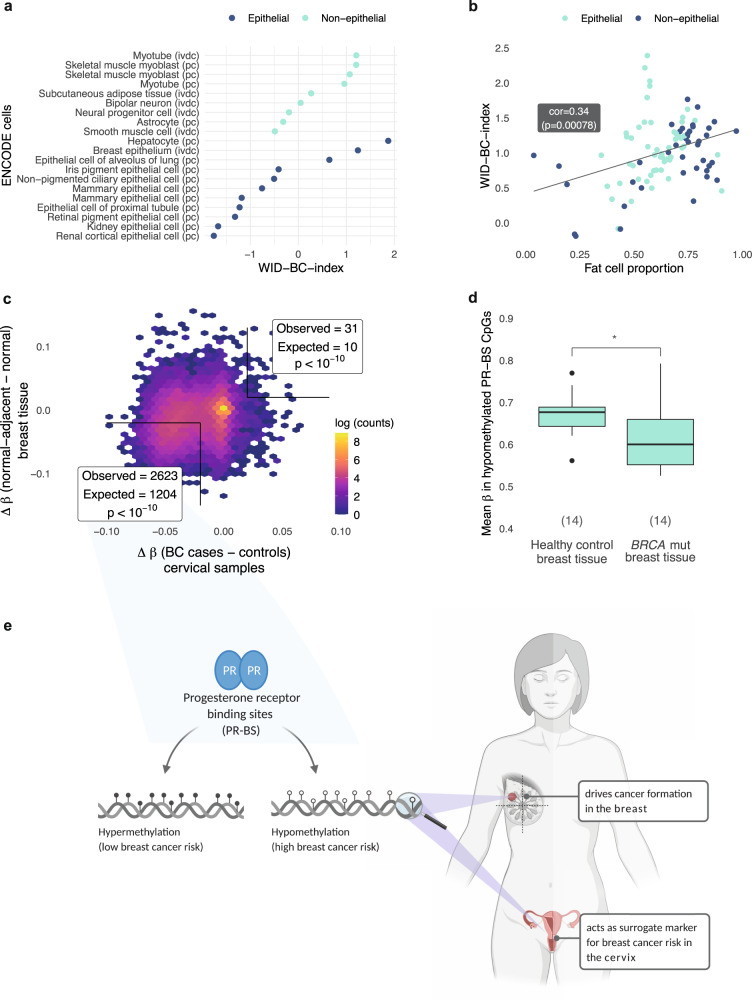
Functional assessment of the WID-BC-index. **a** The WID-BC-index evaluated in ENCODE primary cells (pc) and in vitro differentiated cells (ivdc). **b** The WID-index evaluated in ENCODE tissue samples. Correlation and *p* value assessed using Pearson’s correlation. **c** Difference in mean methylation (Δβ) at progesterone receptor binding site (PR-BS) CpGs between normal-adjacent and normal tissue in breast samples versus the Δβ in breast cancer cases and controls in cervical samples. CpGs with Δβ > 0.02 or Δβ < 0.02 were considered hypo- or hyper-methylated respectively. Observed/expected values and *p* values were assessed using Chi-Squared test: hypomethylated CpGs: *p* < 2.2e-16; hypermethylated CpGs: *p* = 1.349e-11. **d** Mean methylation of the 2,623 hypomethylated PR-BS CpGs in normal breast samples from healthy controls and women with *BRCA* mutation (*p = 0.019 in two-sided Wilcoxon signed-rank test). Values in brackets indicate sample numbers (*n* = 14 healthy controls, 14 *BRCA* mutation carriers). Box plots correspond to standard Tukey representation, with boxes indicating mean and interquartile range, and lines indicating smallest and largest values within 1.5 times of the 25^th^ and 75^th^ percentile, respectively. Dots indicate outlier values. **e** Schematic illustration created using Biorender. Hypomethylation at PR-BS CpGs is associated with an increased breast cancer risk and can be detected in breast tissue at risk and in cervical samples. Source data are provided as a Source Data file.

In order to assess whether the WID-BC-index is reflective of a cell-specific program, we analysed all ENCODE samples (Supplementary Table 8) for which EPIC array data were available. We ranked and plotted the WID-BC-index in all primary cell samples and in vitro differentiated cell samples and found a high WID-BC-index in nonepithelial cells (Fig. 6a). The majority of tissue samples contain substantial proportions of fat, as determined by the EpiDISH algorithm, and hence we have plotted the WID-BC-index against the fat content of the respective tissue samples (Fig. 6b). We found a direct correlation (0.34, *p* value < 0.001) between the WID-BC-index and the fat content of the sample irrespective of whether the sample was taken from an epithelial or nonepithelial organ. These findings indicate that the WID-BC-index is reflective of a fat cell program.

### Cervical epithelial cells mirror breast epithelial tissue

Amongst the key drivers for poor prognostic breast cancer^17,20^, progesterone is well known for its lipogenic effect in adipocytes but also breast cells^33–35^. Hence we wanted to assess whether aberrant DNAme at progesterone receptor binding sites (PR-BS)^36,37^ in at-risk breast tissue is mirrored in cervical cells. We assessed the 19,258 PR-BS CpGs which are available on the EPIC array and computed the differences in mean methylation at PR-BS CpGs in 14 normal breast samples from healthy women and 14 normal breast samples from women adjacent to their triple-negative breast cancer. We compared this to the differences in mean cervical PR-BS methylation between healthy controls and breast cancers from our discovery set cervical samples and observed a substantial skew towards PR-BS sites which are hypomethylated in both cervical and breast tissue in at-risk women with a highly significant overlap of 2,623 hypo-methylated CpGs (defined as a methylation difference < −0.02) in both breast and cervical samples (Fig. 6c). Analysis of cellular components revealed equal proportions of epithelial, immune, fat, and fibroblast cells in these samples, suggesting that alterations in methylation are not primarily driven by changes in cellular composition (Supplementary Fig. 1i). Furthermore, we observed a significantly lower mean methylation at these 2,623 hypo-methylated PR-BS CpGs in 14 normal breast tissue samples from women with a *BRCA* mutation compared to the 14 normal breast tissue samples (Fig. 6d).

Progesterone is known to be a major factor driving ER/PR-negative breast cancer by stimulating the secretion of RANKL and other factors in ER+/PR+ luminal mature cells which activate the expansion of ER−/PR− luminal progenitor cells eventually leading to highly aggressive cancer. Hence, the fact that we see a substantial overlap of PR-BSs in cervical samples from women with breast cancer and in normal breast tissue from women with a BRCA mutation is perfectly aligned with the view that overall higher (lifetime) levels of progesterone in women at risk lead to a reduction of methylation in both surrogate (i.e. cervical) and at-risk (i.e. breast tissue and here likely cells like luminal mature cells) which eventually triggers breast cancer (Fig. 6e).

## Discussion

We have identified a cervical DNAme signature, the WID-BC-index, which provides an opportunity to identify women with a poor prognostic primary breast cancer based on a sample, which has no direct anatomical link to the diseased organ. Women in the top quartile of the WID-BC-index have a 15.7 fold increased risk for breast cancer independent of any other risk factors (Table 1).

Our findings suggest that the alterations to the cervical epigenome which are quantified by the index reflect cancer predisposition in other hormone-sensitive tissues of the body rather than the presence of an established breast cancer. Besides the anatomical distance to the cervix which precludes direct detection of tumour by-products, the index works independently of tumour size or nodal metastasis. Hypomethylation of progesterone receptor binding sites in the cervical samples of women with breast cancer mirrored a similar pattern of hypomethylation in at-risk breast tissue (i.e. in normal breast tissue adjacent to triple-negative breast cancer and in normal tissue from women with a *BRCA* mutation) (Fig. 6e). Progesterone is a well-established driver of poor prognostic breast cancers^17^, and its lipogenic effects are consistent with the view that the index reflects a fat cell epigenetic program.

The notion of predisposition is further supported by the fact that the index also identifies women with ovarian (and to some extent endometrial) cancer and those women who have a very high risk of developing both breast and ovarian cancer (i.e. due to a *BRCA1* mutation)—likely at least in part due to cell-nonautonomous factors^38^. The fact that the WID-BC-index is able to identify women who have a *BRCA1* mutation is quite unexpected because the inclusion criteria that we have chosen for this set (i.e. women without risk-reducing surgery and who had not developed a cancer yet) were biased against those carriers with the highest risk.

The lack of evidence for association with stage and nodal status supports the view that the WID-BC-index is not a marker for the advanced disease but rather a surrogate marker associated with the development/presence of breast cancers with poor prognostic features.

Although in the discovery set the proportion of immune cells in the cervical samples of the breast cancer cases was significantly lower and specifically pronounced for monocytes (consistent with observations in peripheral blood samples from women with incident breast cancers^39^), this effect was not essential for the discriminatory capacity of the WID-BC-index, evidenced by the fact that in the validation set no cell-type difference existed between cases and controls. Moreover, by decomposing the index into tissue-specific components we were able to show that the dominant signal is present in epithelial cells, and is also partly present in immune cells. The validation in matched buccal and blood samples confirm the systemic nature of the DNAme signal, although further prospective studies will be required to confirm whether the WID-BC-index can be used as a biomarker of breast cancer risk. Due to the current study design, disease effects cannot entirely be ruled out.

Our findings described here are consistent with data published more than 30 years ago showing that patients with hereditary breast cancer and their first-degree relatives harbour a differentiation defect^40^. We speculate that at least part of this differentiation defect might be triggered by aberrant exposure to progesterone. Progesterone levels fluctuate (i.e. menstrual cycle, pregnancies, oral contraceptive pill use, hormone replacement therapy) and hence for practical reasons it is impossible to quantify progesterone exposure over time using conventional technologies (i.e. serum analysis); DNAme at those DNA sites which are bound by the progesterone receptor might act as a good surrogate marker for overall exposure and thereby reflects breast cancer predisposition.

Considerable effort in the past has shown that by combining SNPs, mammographic density, and epidemiologic risk factors the AUC corresponding to breast cancer prediction can be increased up to 0.68^6^. Due to the inherently fixed nature of the genome and the plastic nature of the epigenome, it is not possible at this time to state whether DNAme in a cervical liquid-based cytology sample truly outperforms the currently available (largely SNP-based) risk-predicting algorithms and how long in advance of breast cancer diagnosis the cancer risk can accurately be predicted.

We have demonstrated that long-term storage of cervical samples in the methanol-based fluid is associated with a substantial decrease in sample quality and thereby a dramatic decrease in the signal-to-noise ratio. Nevertheless, we have demonstrated that despite this technical limitation some of the signal is retained in the higher quality samples and does not seem to vary depending on the time between sample donation and cancer diagnosis.

Side-by-side comparisons assessing various predictors of risk using the same population will be required in the future. Studying sequential population-based cervical liquid-based cytology samples (in which the DNA has been isolated or the cell pellet stored at −80 °C within a few weeks after sample collection) from women who develop a breast cancer several years later will (i) be a prerequisite for assessing whether the actual risk-predictive nature of the WID-BC-index will truly outperform current breast cancer predictive algorithms and (ii) determine whether the index should be implemented in a clinical setting in order to guide preventive and early detective measures. The WID-BC-index has been developed and validated in the European population; the test also needs to be further validated in non-European populations. Whether DNAme profiles assessed in cervical cytology samples, and/or in breast tissue samples, can also act as surrogates for monitoring breast cancer preventive measures will need to be assessed in large-scale prospective clinical trials.

## Methods

### Study design and epidemiological data acquisition

The case-control study presented in this manuscript is a sub-study of the FORECEE (4 C) programme which was conducted as part of a multicentre study involving several recruitment sites in 5 European countries (i.e. the UK, Czech Republic, Italy, Norway, Germany) (Supplementary Table 9) and has ethical approval from UK Health Research Authority (REC 14/LO/1633) and all contributing centres, including the NRES Committee London (UK), Ethics Committee of the General University Hospital, Prague (Czech Republic), Comitato Etico degli IRCSS Instituto Europeo di Oncologia e Centro Cardiologico Monzino (Itality), Regionale Komiteer for Medisinsk og Helsefaglig Forskningsetikk (Norway), and Ethikkommission bei der LMU München (Germany). Participants were aged >18 years. Prior to taking part, each prospective study volunteer was given a Participant Information Sheet, as well as a Consent Form and the rationale for the study was explained. Additional resources, including an explanatory video and further online resources, were also made available. Women diagnosed with breast or ovarian cancer (case) or a non-malignant benign gynaecological condition (control) were approached during outpatient hospital clinics, while women recruited as *BRCA* mutation carriers or healthy volunteers from the general population (control) were approached via outreach campaigns, public engagement in the UK.

It was necessary for us to consider only pre-treatment samples as an essential part of breast cancer treatment is chemotherapy, Gn-RH-analoga or selective estrogen receptor modulators (e.g. Tamoxifen or aromatase inhibitors). Interference at the level of steroid hormones (i.e. even chemotherapy interferes with hormonal regulation as a large proportion of pre-menopausal women stop menstruating during chemotherapy) leads to a substantial change in DNAme^41^ and therefore it is not appropriate to assess DNAme as a risk-predictive factor after breast cancer treatment.

After signing an informed consent, participants completed an epidemiological questionnaire, as well as a feedback form after their participation.

The epidemiological survey was administered via the Qualtrics application on dedicated iPads. The survey contained questions relating to health habits, relevant risk factors, and also made enquiries as to historical health habits, as well as obtaining a thorough medical and obstetric history. Cervical samples were collected at appropriate clinical venues by trained staff and the collection of cervical liquid-based cytology were carried out by a small group of research midwives or physicians with a view to establishing a standard practice. Buccal samples were collected using Copan 4N6FLOQ Swabs, Thermofisher Scientific. Participants were not compensated for participation.

Biological samples were given an anonymous Participant ID Number, which was assigned to the person’s name in a securely stored link file. Women with a current diagnosis of (a) primary breast cancer with poor prognosis features (Grade III and/or T2/3 and/or N1/2 and/or hormone receptor positive) or (b) malignant invasive epithelial ovarian or endometrial cancer and recruited prior to receiving any systemic treatment (chemo- or endocrine or trastuzumab, etc.) or surgery or radiotherapy were eligible as breast, ovarian or endometrial cancer cases respectively. Clinical and epidemiological characteristics of the discovery and external validation sets are presented in Supplementary Tables 1–3.

For the FORECEE discovery set controls were initially matched one-to-one with cases based on menopausal status, age (5 year age ranges where possible), and recruitment centre/country. However, due to an imbalance in the recruitment of cases and controls at some centres, a number of cases were matched on age and menopausal status alone. Cancer histological data was collected post-recruitment either by clinicians directly involved in the diagnosis/treatment of the cancer cases or by a nominated data manager with access to the in-house hospital systems.

### Cervical liquid-based cytology sample collection

Cervical samples were taken at collaborating hospitals and recruitment centres using the ThinPrep system (Hologic Inc., cat #70098-002). Cervical cells were sampled from the cervix using a cervix brush (Rovers Medical Devices, cat #70671-001) which was rotated 5 times through 360 degrees whilst in contact with the cervix to maximise cell sampling. The brush was removed from the vagina and immersed in a ThinPrep vial containing Preserve-cyt fluid and then pushed against the bottom of the vial 10 times to facilitate the release of the cells from the brush into the solution. The sample vial was sealed and stored locally at room temperature. Buccal cells were collected using two Copan 4N6FLOQ Buccal Swabs (Copan Medical Diagnostics, cat #4504 C) by firmly brushing the swab head 5–6 times against the buccal mucosa of each cheek. The swabs were re-capped and left to dry out at room temperature within the sampling tube which contains a drying desiccant. 2.5 ml of venous whole blood was collected in PAX gene blood DNA tubes (BD Biosciences #761165) and stored locally at 4 ˚C. All samples were shipped to UCL at ambient temperature.

### Clinical cytology biobank archival samples

The Clinical Cytology biobank at the Karolinska University Laboratory, Karolinska University Hospital, systematically stores a compacted aliquot of all ThinPrep liquid-based cytology samples taken as part of the organised cervical screening programme in the Stockholm-Gotland capital region^42,43^. This biobank has ethical approval from the Regionala etikprövingsnämnden i Stockholm. The biobank also stores an aliquot from all samples taken on clinical indication in the region’s gynaecological clinics and the catchment area constitutes ~20% of the entire nation. Women who participate in screening are informed in the invitation letter that their sample will be stored, and potentially used for ethically approved research. Information is given on how to opt out of this procedure, should the woman wish not to have her sample stored. Very few women opt out. We linked all LBC samples in the biobank from years 2011 to 2015 to the Swedish National Cancer Registry, to identify all cases of breast cancer that had occurred among women who had at least one sample stored in the biobank (*n* = 325). We used the same register to identify all women who had not been diagnosed with breast cancer within the study period, and from these sampled a 1:1 control set of healthy women frequency-matched on age and sample year (*n* = 351, for a total of 676 samples). From each vial, 100 µL of biobanked ThinPrep material was retrieved and submitted to UCL for extraction and methylation analyses. Ultimately, 599 samples passed QC and had sufficient phenotypic information available and were used as a further external validation set.

### Breast tissue samples

We have analysed an independent set of breast tissue samples containing a total of 42 breast samples from premenopausal women aged 19–54 years (Supplementary information): normal breast tissue from 14 women who underwent cosmetic breast operations, normal breast tissue from women who underwent prophylactic mastectomies due to a *BRCA1* (*n* = 9) or a *BRCA2* (*n* = 5) mutation (Supplementary Table 10), and 14 normal samples from women who underwent surgery for triple-negative breast cancer (tissue adjacent to the cancer was collected). All samples were collected fresh from theatre and samples processed within 1 hr of surgical excision. Fresh samples were frozen rapidly in Liquid Nitrogen and stored at −80 °C. Ethical approval was obtained from the NRES Committee East of England (reference number 15/EE/0192). All patients provided written informed consent.

### Sample processing and DNA extraction

When preparing for sample storage in the laboratory, cervical samples were poured into 50 ml Falcon tubes and left to sediment at room temperature for 2 h. One mL wide bore tips were then used to transfer the enriched cellular sediment into a 2 mL vial. The cervical sediments were washed twice with PBS, lysed, and stored temporarily at −20 ˚C ahead of extraction. The Copan 4N6FLOQ Buccal Swabs were cut and lysed sequentially in the same aliquot of lysis buffer prior to temporary storage at −20 ˚C ahead of extraction. Whole blood samples were simply held transiently at −20 ˚C until DNA extraction. DNA was extracted from whole blood, cervical and buccal tissue lysates on a Hamilton Star liquid handling platform using the Nucleo-Mag Blood 200ul kit (Macherey Nagel, cat #744501.4) with prior modifications for optimal lysis of cervical cell pellets and paired buccal swabs. For breast tissues, DNA was extracted from up to 40 mg of tissue using the Lipid Tissue kit from Macherey Nagel (cat # 740471.50), and the manufacturer’s instructions were followed. DNA concentration and quality absorbance ratios were measured using Nanodrop-8000, Thermoscientific Inc. Extracted DNA was stored at −80 ˚C until further analysis.

### DNA methylation array analysis

Cervical, buccal and breast tissue DNA was normalised to 25 ng/µL and 500 ng total DNA was bisulfite modified using the EZ-96 DNA Methylation-Lightning kit (Zymo Research Corp, cat #D5047) on the Hamilton Star Liquid handling platform. Eight µL of modified DNA was subjected to methylation analysis on the Illumina InfiniumMethylation EPIC BeadChip (Illumina, CA, USA) at UCL Genomics according to the manufacturer’s standard protocol.

### Methylation analysis

All methylation microarray data were processed through the same standardised pipeline. Raw data was loaded using the R package minfi, version 1.36.0. Any samples with median methylated and unmethylated intensities <9.5 were removed. Any probes with a detection *p* value > 0.01 were regarded as failed. Any samples with >10% failed probes, and any probes with >10% failure rate were removed from the dataset. Beta values from failed probes (approximately 0.001% of the dataset) were imputed using the impute.knn function as part of the impute R package, version 1.62.0.

Non-CpG probes (2932), SNP-related probes as identified by Zhou et al.^44^ (82,108), and chrY probes were removed from the dataset. An additional 6102 previously identified probes that followed a trimodal methylation pattern characteristic of an underlying SNP were removed.

Background intensity correction and dye bias correction was performed using the minfi single sample preprocessNoob function. Probe bias correction was performed using the beta mixture quantile normalisation (BMIQ) algorithm in the ChAMP package, version 2.18.3.

The fraction of immune cell contamination, and the relative proportions of different immune cell subtypes in each sample, were estimated using the EpiDISH package, version 2.6.1, using the epithelial, fibroblast and immune cell reference dataset. The top 1000 most variable probes (ranked by standard deviation) were used in a principal component analysis. Statistical tests were performed in order to identify any anomalous associations between plate, sentrix position, date of array processing, date of DNA creation, study centre, immune contamination fraction, age, type (case versus control) and the top ten principal components. Finally, two-thirds of the discovery dataset was randomly selected for use as the training dataset and the remaining third was allocated to the internal validation dataset. This split was carried out once, and the same training and validation sets were used in all subsequent analyses.

A total of 113 samples were downloaded from the ENCODE database (https://www.encodeproject.org/; see Supplementary Table 8). BMIQ was applied to these samples after using minfi to extract beta values.

### Statistical analyses

All statistical analyses were carried out in R 4.0.2 and all significance testing was conducted two-sided. Areas under the curve of the receiver operating characteristic (AUROCs) and corresponding 95% confidence intervals were calculated using the pROC R package, version 1.18.0.

### Classifier development

Contamination by immune cells presented a challenge with respect to the identification of differentially methylated positions (DMPs) as differential methylation that occurred solely in epithelial cells was diminished in samples with high immune cell proportion and vice versa. In order to overcome this, we linearly regressed the beta values on immune cell proportion for each CpG site, the linear models being fitted to cases and controls separately. The intercept points at immune cell proportion = 0 were used as estimates of mean beta values in cases and controls in a pure epithelial cell population. The difference between these intercept points provided a delta-beta estimate in epithelial cells. The difference between intercept points at immune cell proportion = 1 provided immune cell delta-beta estimates. A list of ranked CpGs was produced according to delta-beta estimates in epithelial cells. Age was included as an additional variable when linearly regressing on immune cell proportion.

The R package glmnet, version 2.0–18, was used to train classifiers with a mixing parameter value of alpha = 0 (ridge penalty) and alpha = 1 (lasso penalty) with binomial response type. Data from the training dataset were used to fit the classifiers. A ranked list of CpGs was generated by taking the CpG with the largest epithelial delta-beta, followed by the CpG with the largest immune delta-beta, followed by the next largest epithelial delta-beta and so forth (any duplicates were removed). The top n CpGs from the list of ranked CpGs were used as inputs to the classifier. Ten-fold cross-validation was used inside the training set by the cv.glmnet function in order to determine the optimal value of the regularisation parameter lambda. The AUC was used as a metric of classifier performance which was evaluated on the internal validation dataset as a function of n, the number of CpGs used as inputs during training. The maximum value of n was 30,000.

The optimal classifier was selected based on the highest AUC obtained in the internal validation dataset. Once the optimal number of inputs was determined, the training and internal validation datasets were combined and the classifier was refitted using the entire discovery dataset with alpha and lambda fixed to their optimal values. This finalised classifier was then applied to the external validation dataset and the corresponding AUC was computed.

Denoting the top n CpGs as ${\beta }_{1},\ldots ,{\beta }_{n}$ and the regression coefficients from the trained classifier as ${w}_{1},\ldots ,{w}_{n}$ then WID-BC-index = $\mathop{\sum }\nolimits_{i=1}^{n}({w}_{i}{\beta }_{i}-\mu )/\sigma$ where μ and σ are defined as the mean and standard deviation of the quantity $\mathop{\sum }\nolimits_{i=1}^{n}{w}_{i}{\beta }_{i}$ in the training dataset (that is, the index is scaled to have zero mean and unit standard deviation in the training dataset).

The cv.glmnet function in the glmnet R function was used to compute an estimate of the pseudo-R^2^ (using the dev.ratio quantity). The Brier score was computed using the brier.score function in the iterativeBMA R package, version 1.42.

A calibration curve was generated using the val.prob function in the rms R package, version 6.1-1 (Supplementary Note 1). The internal validation set was used to recalibrate the index before computing the calibration curve corresponding to the recalibrated index in the independent external validation dataset. Recalibration was done by using the internal validation dataset to fit the following logistic model *Y* = *α* + *β* $*$ *index*, where $Y={{\log }}(\frac{p}{1-p})$ and $p=\frac{{e}^{{index}}}{1+{e}^{{index}}}$ is the probability of being a case. The parameters α and β are termed the calibration intercept and slope respectively. The index is rescaled by multiplying by the calibration slope and then adding the calibration intercept as described by Van Calster et al^45^.

### Development of the TCGA-BC-index

Illumina 450 K Array Methylation data from primary tumours (*n* = 791) and normal tissue (*n* = 96) was downloaded from TCGA using the TCGAbiolinks R package, version 2.16.4, extracting all primary tumour and normal tissue samples in the TCGA-BRCA project [https://portal.gdc.cancer.gov/projects/TCGA-BRCA] for which Illumina 450k Methylation Data was available. The data were randomly split into a training and a testing set (70−30%). The TCGA-BC-index was developed by identifying the top differentially methylated CpGs and classifier development as outlined above. The final TCGA-BC-index consisted of 31 CpGs and was assessed in the testing set as well as the 3 C discovery and validation set.

### Decomposition of index

We aimed to estimate how much variability across the 29,000 CpGs in the WID-BC-index could be attributed to epithelial cells or immune cells. An example of a CpG with high variability in epithelial cells and low variability in immune cells is given in Fig. 2c. For each CpG we applied the following model. We assumed that the epithelial beta values follow a beta distribution ${Beta}(\beta |{a}_{0},{b}_{0})$ with shape parameters a_0_ > 0 and b_0_ > 0, and that immune beta values followed ${Beta}(\beta |{a}_{1},{b}_{1})$ with shape parameters a_1_ > 0 and b_1_ > 0. We assumed that each sample is a combination of epithelial and immune cells and that ${\rho }_{i}\in [{{{{\mathrm{0,1}}}}}]$ is the proportion of immune cells in sample $i,i=1,\ldots ,N.$ The quantities ρ_i_ were obtained from the EpiDISH algorithm. The following log likelihood function (1) was numerically optimised with respect to ${a}_{0},{b}_{0},{a}_{1},{b}_{1}$:

1 $$L{\left({a}_{0},{b}_{0},{a}_{1},{b}_{1}\right)}=-\frac{1}{N}{\mathop{\sum}\limits_{i=1}^{N}}{\mathrm log}[(1-{\rho }_{i}){Beta}\left({\beta }_{i}{|}{a}_{0},{b}_{0}\right)+{\rho }_{i}{Beta}({\beta }_{i}{{|}}{a}_{1},{b}_{1})]$$

and the variance of the epithelial and immune beta distributions were used as estimates of epithelial and immune variance. CpGs were classified as epithelial, shared, or immune as shown in Fig. 2g. This resulted in the following decomposition formula of the index (2):

2 $${{\mbox{WID-BC-index}}}=\mathop{\sum }\limits_{{i\in I}_{{epithelial}}}\frac{{w}_{i}{\beta }_{i}-\mu }{\sigma }+\mathop{\sum }\limits_{{i\in I}_{{shared}}}\frac{{w}_{i}{\beta }_{i}-\mu }{\sigma }+\mathop{\sum }\limits_{{i\in I}_{{immune}}}\frac{{w}_{i}{\beta }_{i}-\mu }{\sigma }$$

where the coefficients w_i_ and the quantities μ and σ are the same as defined above.

### Definition of PR-BS CpGs

Progesterone receptor binding site (PR-BS) CpGs were defined based on published progesterone receptor ChIP-Seq datasets identified as high quality from the Cistrome Database^36,37^. Filtered progesterone binding site (GSE40724 [https://www.ncbi.nlm.nih.gov/geo/query/acc.cgi?acc=GSE40724])^37^ or raw data (GSE68355 [https://www.ncbi.nlm.nih.gov/geo/query/acc.cgi?acc=GSE68355])^36^ were accessed from GEO using the GEOquery R package, version 2.58.0. Raw data were preprocessed to filter for fold change enrichment >10 and FDR < 5% (using the “fold_enrichment” and “FDR (%)” variables in the raw data files), and consensus binding sites were defined as occurring in two out of three replicates. CpGs in these ChIP-Seq peaks were identified using the Illumina EPIC array Probe manifest. PR-BS CpGs were defined as CpGs detected in both PR ChIP-Seq datasets (*n* = 19,258).

### Analysis of PR-BS CpGs in breast and cervical samples

For each of the 19,258 PR-BS CpGs we defined ∆β as the difference in mean beta value between breast cancer cases and controls (in cervical tissue) and normal-adjacent and normal tissue (in breast tissue). We then counted the number of observed CpGs with ∆β < -0.02 in both breast and cervical datasets (2,623 in total) and similarly the number with ∆β>0.02 in both datasets (31 in total). The threshold of 0.02 was prespecified based on typical observed differences in cervical samples, although the main focus of this analysis was to assess the common directionality of methylation in cervical samples and breast tissue rather than compare absolute methylation differences. To test whether we observed significantly more overlapping CpGs than expected, a contingency table of PR-BS CpGs either hypo- (defined as ∆β < −0.02) or hypermethylated (defined as ∆β > 0.02) in one or both datasets was created and p-values were generated using the Chi-Squared test. The expected proportion of overlapping CpGs was calculated as the proportion of PR-BS CpGs hypomethylated in cervical samples multiplied by the proportion of PR-BS CpGs hypomethylated in breast samples. The same approach was applied to hypermethylated PR-BS CpGs.

We calculated the mean beta values of the 2,623 overlapping hypomethylated CpGs in 14 normal breast tissue samples from healthy controls and 14 *BRCA* mutation carriers. Statistical analysis was carried out using Wilcoxon tests (paired for before-after comparison). Figure 6e was created with BioRender.

### Methylation quantitative loci analysis

mQTL data was obtained from the supplementary information of a recent publication by Ho et al.^32^. The 822 probes at loci associated with breast cancer risk were extracted and those present in the WID-BC-index (*n* = 78) were removed from the WID-BC-index for assessment of their influence on the performance.

### SNP genotyping, QC and imputation

In total, 318 breast cancer case subjects and 850 controls from the discovery set (Table 1) were taken forward for genotyping using an Illumina 650k Infinium Global Screening Array (GSA). Whole blood DNA was normalised to 75 ng/µL and a total of 300 ng applied to the Infinium Global Screening Array – 24 V2 (Illumina, CA, USA) at UCL Genomics according to the manufacturer’s standard protocol.

One control subject from this cohort failed to genotype. Genotype calling was performed using GenomeStudio, with genetic variants found to be clustering poorly being removed from further analyses. For duplicate genetic variant pairs, the variant within each pair with the lowest calling and clustering score was excluded. Autosomal SNPs were used in subsequent QC and PRS analyses (except for checks for sex mismatches, where the X chromosome was used to infer sex).

General subject and single nucleotide polymorphism (SNP) quality control (QC) was performed using PLINK version 1.9^46^. Three breast cancer cases and eight controls with a call rate less than 95% were excluded. One breast cancer case and three controls were further removed due to genetically inferred sex not being female. Genetic variants with a missing genotype rate greater than 5%, minor allele frequency (MAF) less than 1% or a significant departure from Hardy-Weinberg equilibrium (*p* value < 5 ×10^−6^) were excluded.

KING^47^, a relatedness inference algorithm, was used to identify duplicate/monozygotic twin or first-degree relative pairs. One control subject pair was identified as being a duplicate/monozygotic twin pair, and nine control pairs were inferred to be first-degree relatives. The subject within each related pair with the lowest call rate was excluded. After performing QC, 314 breast cancer case subjects, 816 controls and 479,105 variants were retained in the SNP discovery sample.

Non-European subjects were identified by plotting the top two principal components, generated using GCTA version 1.26.0, for the SNP discovery samples and 270 HapMap phase II release 23 samples (CEU, YRI, JPT and CHB individuals) downloaded in PLINK-formatted binary files from http://zzz.bwh.harvard.edu/plink/res.shtml. Subjects found not to cluster around HapMap European samples were excluded from further analyses. After excluding non-European subjects, 305 breast cancer cases and 754 controls were retained in the SNP discovery sample.

Using the Michigan Imputation Server^48^ and 1000 Genomes Phase 3 reference panel, the SNP discovery dataset went through further QC before being phased (Eagle2) and imputed. Variants where strand, allele, genetic position, or allele frequencies were not concordant with the 1000 Genomes Phase 3 reference panel were removed before phasing and imputation using Strand Tools.

After imputation, exclusion of variants with imputation *R*^2^ < 0.5 and removal of variants observed to have three or more alleles, 303 of the 313 SNPs used by Mavaddat et al.^31^ to develop a 313 SNP breast cancer polygenic risk score (PRS) were successfully imputed. We constructed a breast cancer PRS for each subject in the discovery set, such that the PRS is equal to (3):

3 $$PR{S}_{j}=\mathop{\sum }\limits_{i=1}^{303}\widehat{{\beta }_{i}}{x}_{ij}$$

where, ${\hat{\beta }}_{i}$ is the log odds ratio for the *i*-th SNP taken from publicly available Oncoarray summary association results^49^ (combined Oncoarray, iCOGs and BCAC overall breast cancer beta values) and x_ij_ is the number of copies of the effect allele present in each discovery set subject subjected to genotyping. Scores were generated using PLINK version 1.9.

### Reporting summary

Further information on research design is available in the Nature Research Reporting Summary linked to this article.

## Supplementary information

Supplementary Information

Peer Review File

Reporting Summary

## Data Availability

Previously published data were accessed from ENCODE (accession codes listed in Supplementary Table 3), GEO (GSE40724) and GSE68355), and TCGA (TCGA-BRCA project [https://portal.gdc.cancer.gov/projects/TCGA-BRCA]). Raw DNAme and SNP data generated in this study have been deposited in the European Genome-phenome Archive (EGA) database under the study accession codes EGAS00001005055 (breast cancer cervical and buccal methylation methylation and PRS SNP data), EGAS00001005070 (breast tissue methylation), EGAS00001005045 (ovarian cancer cervical methylation), EGAS00001005033 (endometrial cancer cervical methylation data), and EGAS00001005626 (matched methylation data from cervical, buccal, and blood samples from controls and BRCA1/2 mutation carriers). The raw data are available under restricted access due to patient confidentiality and privacy laws. Access can be obtained by formal application to the relevant Data Access Committee via EGA and signing of a Data Access Agreement. Source data for this paper are provided under https://github.com/chiaraherzog/WID-BC-source-data or available alongside this manuscript. Source data are provided with this paper.

## Source data

Source Data

## Code availability

The code to calculate the WID-BC-index is available as an R package under Github repository https://github.com/chiaraherzog/WID.BC^50^. Use of this code is permitted for research use only.
