# Supplementary material for: The WID-BC-index identifies women with primary poor prognostic breast cancer based on DNA methylation in cervical samples
Source: Nat Commun. 2022 Feb 1;13:449. doi: 10.1038/s41467-021-27918-w (PMC8807602; doi:10.1038/s41467-021-27918-w)
Supplement: Supplementary file 1 — Supplementary Information [file 41467_2021_27918_MOESM1_ESM.pdf]

Supplementary files to "The WID-BC-index identifies women with primary poor prognostic breast cancer based on DNA methylation in cervical samples"

James E. Barrett*, Chiara Herzog*, Allison Jones, Olivia C. Leavy, Iona Evans, Susanne Knapp, Daniel Reisel, Tatiana Nazarenko, Yoo-Na Kim, Dorella Franchi, Andy Ryan, Joanna Franks, Line Bjørge, Michal Zikan, David Cibula, Nadia Harbeck, Nicoletta Colombo, Frank Dudbridge, Louise Jones, Karin Sundström, Joakim Dillner, Angelique Flöter Rådestad, Kristina Gemzell-Danielsson, Nora Pashayan, Martin Widschwendter.

* contributed equally

This file contains:

Supplementary Methods

Supplementary Note 1

Supplementary Figures (1-7) & Tables (1-10)

Supplementary References

Supplementary Methods

Signal-to-noise inference on the Illumina EPIC array

Illumina EPIC array probe design

The Illumina EPIC array consists of two probe types. Type I probes come in pairs with one designed to bind to methylated DNA (after bisulfite modification) and the second designed for unmethylated DNA. Type I pairs measure signal using either the red or green intensity channels. If a type I probe uses the red channel, for instance, then green intensities from that pair will be due to background fluorescence and can be used as an out-of-band estimate of background green intensity. We used 184,454 out-of-band green intensities from type I probes to estimate the distribution of background green intensity, and similarly 99,908 out-of-band red intensities.

In contrast, type II probes consist of single probes. Green intensity corresponds to the methylated signal and red to the unmethylated signal. It is generally not possible to use type II probes to estimate background intensity. Type II probes tend to have a lower CG content and a considerably lower signal intensity in comparison to type I probes. In Supplementary Figure 6A we compared intensities from the Swedish cytology biobank dataset and discovery dataset. The biobanked samples suffered from a lower overall signal intensity leading to lower signal-to-noise ratios in these samples.

Background definitions

Let ϵ_r and ϵ_g denote background red and green background intensities and let M_s and U_s denote the true methylated and unmethylated intensities. For type II probes assume the observed methylated intensity is $M = M_s + \epsilon_g$ (methylated signal in green channel) and $U = U_s + \epsilon_r$(unmethylated signal in red channel). Define the true beta value as $\beta_s = M_s/(M_s + U_s)$ the background beta value as $\beta_\epsilon = \epsilon_g/(\epsilon_g + \epsilon_r)$, and the signal-to-noise ratio as $\rho = (M_s + U_s)/(\epsilon_g + \epsilon_r)$. The observed beta value is given by

$$\begin{aligned}\beta &= \frac{M}{M+U} \\ &= \frac{M_s + \epsilon_g}{M_s + U_s + \epsilon_g + \epsilon_r} \\ &= \frac{\rho\beta_s + \beta_\epsilon}{1+\rho} \qquad (1)\end{aligned}$$

If ρ → 0 then β → $\beta_\epsilon$and if ρ → ∞ then β → β_s as expected.

Lower signal-to-noise reduces the dynamic range of beta values

Lower values of ρ will distort beta values away from 0 and 1. Supplementary Figure 6b shows a simulated CpG site with parameters fixed to ϵ_r = 500 and ϵ_g = 200 (hence β_ϵ = 0.29) and true intensities ranging from 0 to 20,000. The lines correspond to different beta values which become 'squashed' towards the background beta values at low values of ρ. We expect that in samples with lower signal-to-noise ratios the distribution of type II beta values will have a smaller range. Supplementary Figure 6c shows an example of a high signal-to-noise sample in blue and a low-quality sample in red.

Rationale
Reduced red and green signal intensities will lead to a distortion in the distribution of beta values. The distribution of beta values will have a smaller range with modes 'squashed' further away from zero and one. This effect will be most pronounced in type II probes as these probes have a considerably lower signal intensity in comparison to type I probes (Supplementary Figure 6a). Our analysis will therefore focus on type II probes only.

At each probe the measured intensities are a combination of the true signal plus the background intensity. The background intensities are not directly measurable. We reasoned that in samples with a lower signal-to-noise ratio the overall distribution of raw type II beta values will have a diminished range, and this can be used as the basis for inferring the signal-to-noise ratio for each sample.

Inference of signal-to-noise ratios
Beta values typically follow a bimodal distribution with one mode close to zero (the 'unmethylated' mode, denoted by $\beta_0(\rho)$) and a second mode close to one (the 'methylated' mode, denoted by $\beta_1(\rho)$). See Supplementary Figure 6c for an example. We assume that in each sample the location of these modes follows the same parametric form as equation (1). Predicted modes are

$$\beta_0(\rho) = \frac{\rho\beta_s^0 + \beta_\epsilon}{1+\rho} \quad (2)$$

$$\beta_1(\rho) = \frac{\rho\beta_s^1 + \beta_\epsilon}{1+\rho}. \quad (3)$$

For a given sample with observed modes β_0^{obs} and β_1^{obs} we estimate ρ via

$$\mathrm{argmin}_\rho \left[(\beta_0(\rho) - \beta_0^{obs})^2 + (\beta_1(\rho) - \beta_1^{obs})^2\right].$$

The parameter β_ϵ is fixed to a value of 0.33 based on the median out-of-bag type I red and green background intensities in the discovery set. The parameters β_s^0 and β_s^1 are set to 0.02 and 0.97 respectively based on the median unmethylated and methylated modes of type I probes in the discovery set (type I probes have considerably higher red and green values and hence serve as the 'true' locations of the modes).

Application to the discovery set and the biobank sample set
The parameters β_ϵ, β_s^0, and β_s^1 were fixed prior to analysis of both the discovery and biobanked samples. We observed an excellent model fit after inferring optimal ρ values for each sample (Supplementary Figure 6d and Supplementary Figure 6e).

Supplementary Note 1

Assessment of performance

The pseudo-R^2 for the glmnet model fitted to the training dataset is 0.99 (this is analogous to the R^2 goodness-of-fit measure, the proportion of total variance explained by the model, in linear regression). The Brier score corresponding to the WID-BC-index in the internal validation set was 0.16 (the Brier score is the mean square difference between predicted probabilities and actual outcomes, it takes a value between 0 and 1 with smaller values corresponding to better predictors).

In addition, a calibration curve of the WID-BC-index applied to the external validation dataset was plotted (Supplementary Figure 7). The index was first rescaled in order to optimise calibration using the internal validation dataset. The calibration curve shows that the WID-BC-index is well calibrated, with the predicted probabilities only slightly higher than the actual probabilities (i.e. the index tends to slightly overestimate the probability of being a case).

Association with epidemiological and clinical factors

A separate classifier was trained on samples from the internal validation set using the WID-BC-index, PRS, age, age at menarche, parity, age at first pregnancy, menopause age, BMI, oral contraceptive pill use, hormone replacement therapy use, smoking, and first degree relatives with breast cancer as input variables. A logistic regression model was trained on 192 controls and 78 breast cancers and subsequently evaluated in 88 controls and 29 breast cancers. We found that adding these additional variables resulted in an AUC of 0.84 and therefore did not sufficiently improve performance over the index alone. A model based on PRS and epidemiological factors only resulted in an AUC of 0.74.

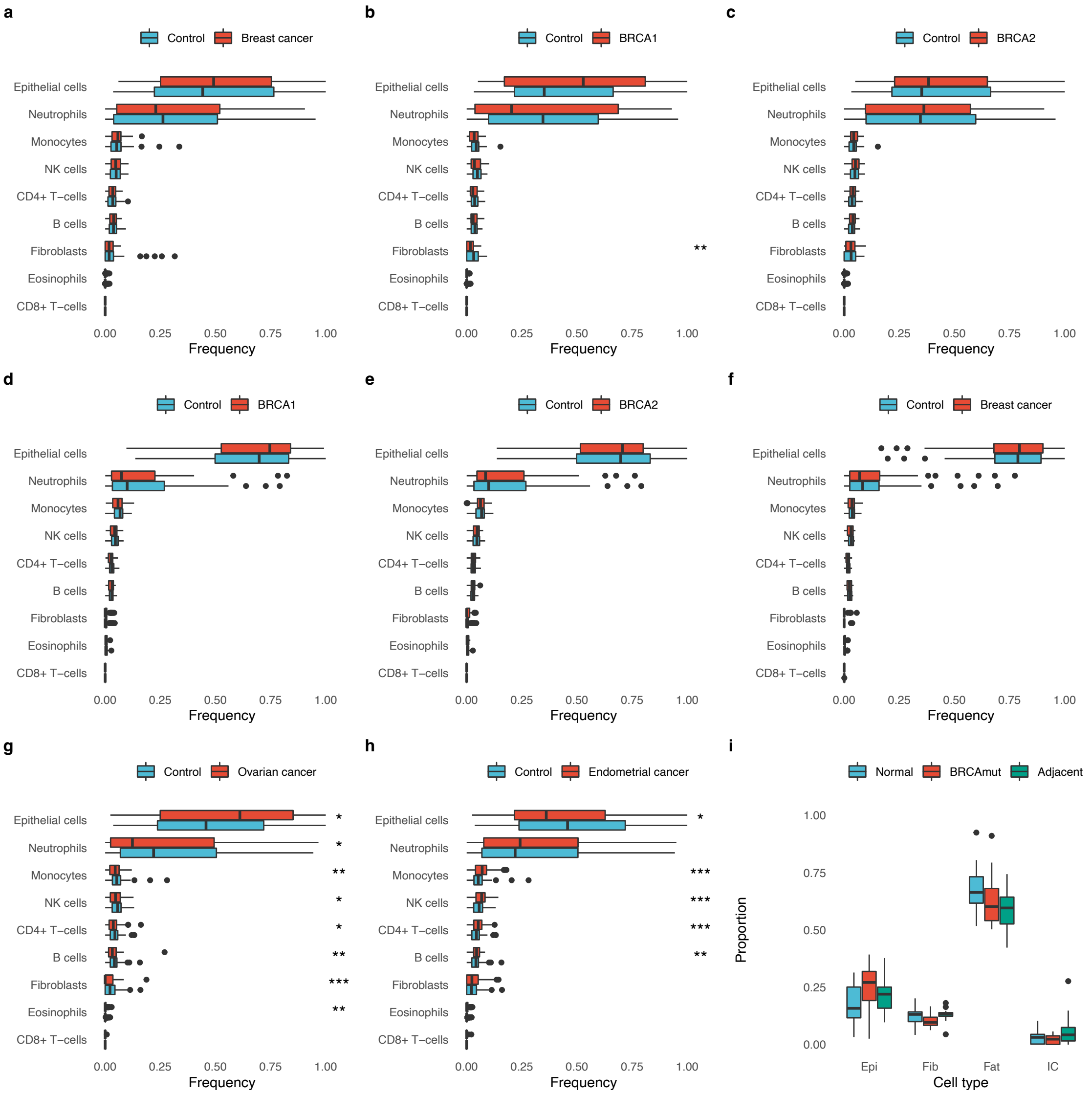

Supplementary Figure 1. **Cell type composition.** **a** Distribution of different cell types as estimated using the HEpiDISH algorithm in cervical samples of the the external validation dataset (n=225 controls, 113 breast cancer cases), **b** cervical samples from *BRCA1* (n=114 controls, 57 *BRCA1* mutation carriers; ** p=0.003 in Wilcoxon signed-rank test) or **c** *BRCA2* mutation carriers (n=114 controls, 53 *BRCA2* mutation carriers), **d** buccal samples from *BRCA1* (n=115 controls, 57 *BRCA1* mutation carriers) or **e** *BRCA2* mutation carriers (n=115 controls, 50 *BRCA2* mutation carriers), **f** matched buccal samples from the breast cancer internal validation dataset (n=69 controls, 66 breast cancer cases), **g** ovarian cancer samples (n=297 controls, 242 ovarian cancer cases; * p<0.05, ** p<0.01, *** p<0.001 in Wilcoxon signed-rank test; exact p-values: epithelial cells, p=0.012; neutrophils, p=0.012; monocytes, p=0.001; NK cells, p=0.017; CD4+ T cells, p=0.014; B cells, p=0.004; fibroblasts, p=0.00007, eosinophils, p=0.009), **h** endometrial cancer samples (n=297 controls, 217 endometrial cancers; * p<0.05, ** p<0.01, *** p<0.001 in Wilcoxon signed-rank test; exact p-values: epithelial cells, p=0.029; monocytes, p=0.0000006; NK cells, p=0.0000007; CD4+ T cells, p=0.000001; B cells, p=0.001), and **i** fresh breast tissue samples (n=14 per group). Box plots correspond to standard Tukey representation, with boxes indicating mean and interquartile range, and lines indicating

smallest and largest values within 1.5 times of the 25th and 75th percentile, respectively. Dots indicate outlier values. Source data are provided as a Source Data file.

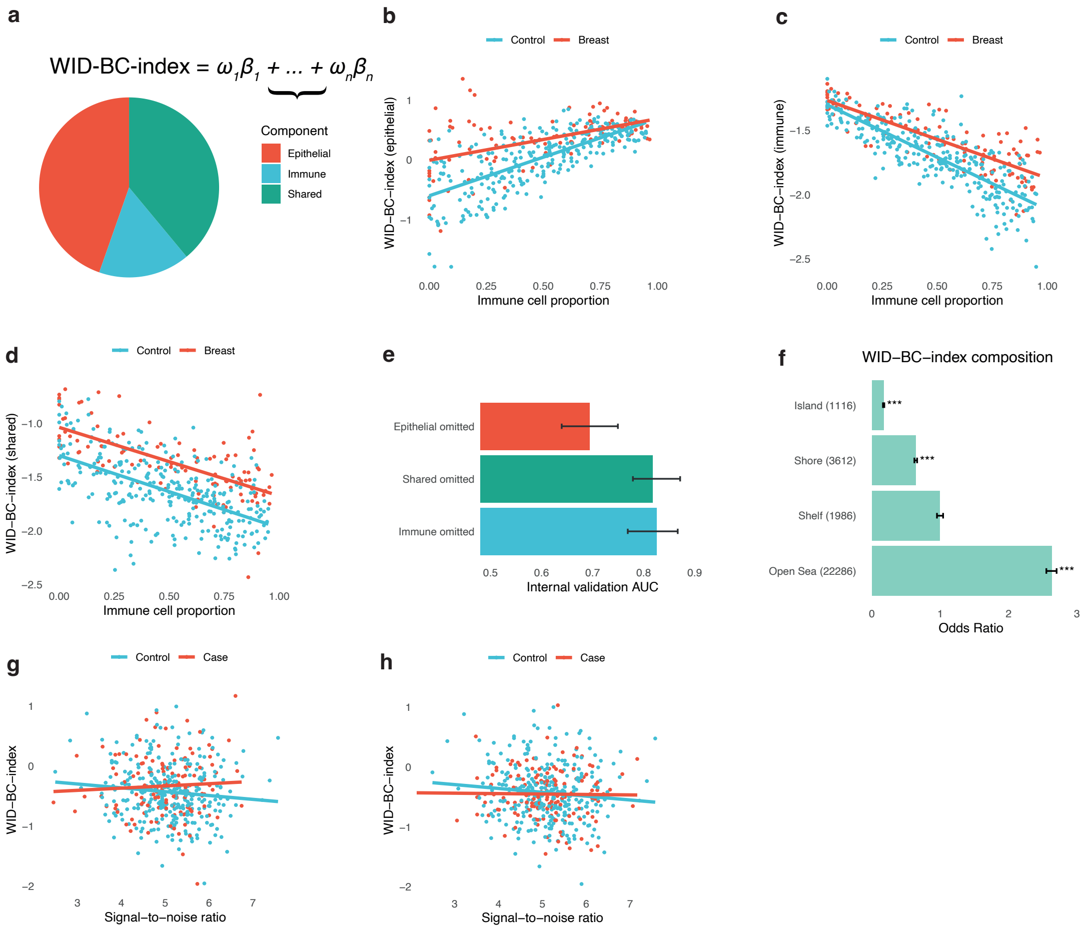

Supplementary Figure 2. **Decomposition of WID-BC-index.** **a** The proportion of variance, weighted by the absolute coefficient values from the WID-BC-index, corresponding to each component of the index. **b** epithelial, **c** immune, and **d** shared subcomponents of the WID-BC-index in the internal validation dataset. **e** Performance of the WID-BC-index in the internal validation set after omitting each subcomponent. Error bars indicate 95% confidence interval. **f** Odds ratios computed corresponding to the genomic annotation of the 29,000 CpGs comprising the WID-BC-index (when compared to the 776,725 CpGs in the dataset; ***, p<2.2e-16 in Chi-Squared test). Error bars indicate 95% confidence interval. **g** The WID-BC-index versus inferred signal-to-noise ratio in poor prognostic breast cancer cases and controls; **h** the WID-BC-index versus inferred signal-to-noise ratio in non-poor prognostic breast cancer cases and controls. Source data are provided as a Source Data file.

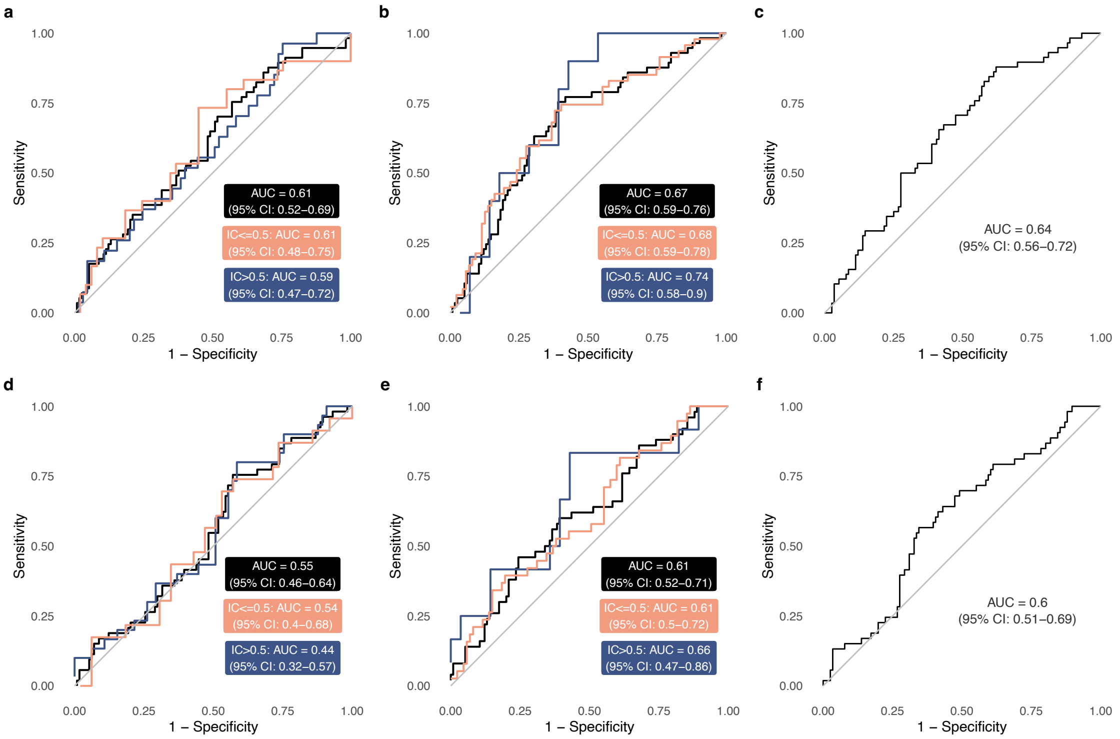

Supplementary Figure 3. **Performance of the WID-BC-index in *BRCA* mutation carriers.** **a** ROC curves corresponding to the WID-BC-index discriminating between *BRCA1* carriers and controls in cervical (n=114 controls, n=57 *BRCA1* mutation carriers), **b** buccal (n=115 controls, n=57 *BRCA1* mutation carriers), and **c** blood samples (n=116 controls, n=58 *BRCA1* mutation carriers), and between *BRCA2* carriers and controls in **d** cervical (n=114 controls, n=53 *BRCA2* mutation carriers), **e** buccal (n=115 controls, n=50 *BRCA2* mutation carriers), and **f** blood samples (n=116 controls, n=53 *BRCA1* mutation carriers). Source data are provided as a Source Data file.

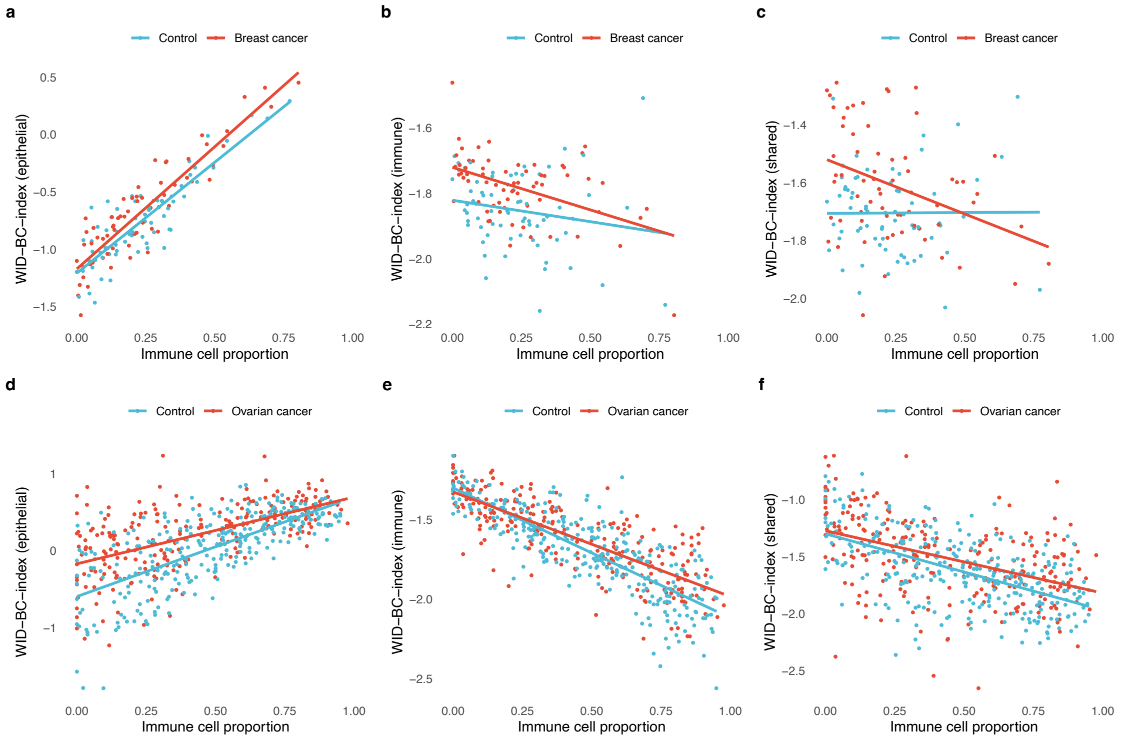

Supplementary Figure 4. **Analysis of epithelial, immune, and shared subcomponents.** **a** Epithelial, **b** immune, and **c** shared subcomponents of the WID-BC-index in matched buccal samples from the breast cancer internal validation dataset. **d** Epithelial, **e** immune, and **f** shared subcomponents of the WID-BC-index in ovarian cancer cases and healthy controls. Source data are provided as a Source Data file.

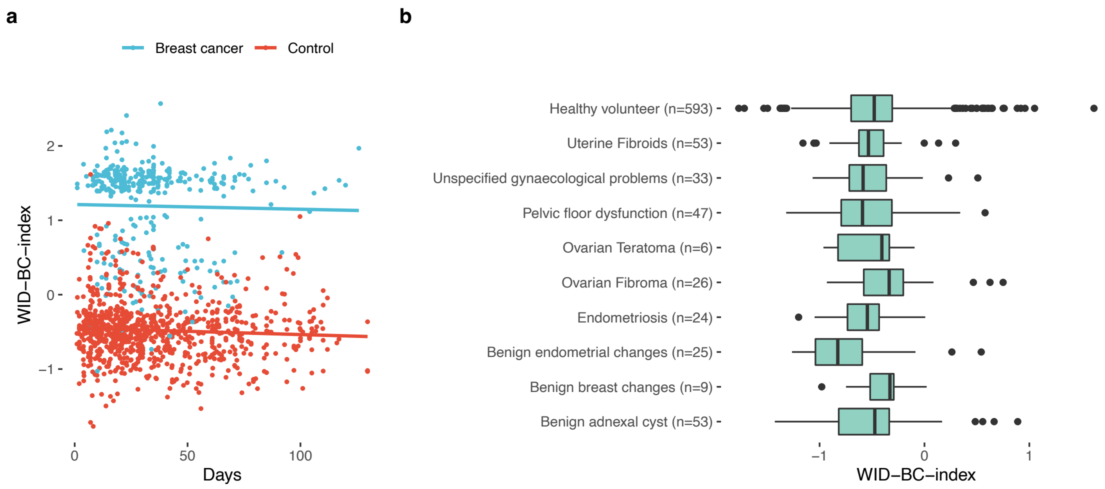

Supplementary Figure 5. **Distribution of WID-BC-index in respect to storage and in healthy volunteers versus patients with benign gynaecological diseases a** The WID-BC-index is independent of the time between sample collection and processing (discovery set). **b** Distribution of the WID-BC-index in controls that volunteered from the general population and women that presented at hospitals for benign women-specific conditions (discovery set). Numbers in brackets indicate sample size (n=593 healthy volunteers, n=53 patients with uterine fibroids, n=33 individuals with unspecified gynaecological problems, n=47 patients with pelvic floor dysfunction, n=6 and n=26 patients with ovarian teratoma and fibroma, respectively, n=24 endometriosis patients, n=25 benign endometrial changes, n=9 individuals with benign breast changes, and n=53 patients with benign adnexal cysts). Box plots correspond to standard Tukey representation, with boxes indicating mean and interquartile range, and lines indicating smallest and largest values within 1.5 times of the 25th and 75th percentile, respectively. Dots indicate outlier values. Source data are provided as a Source Data file.

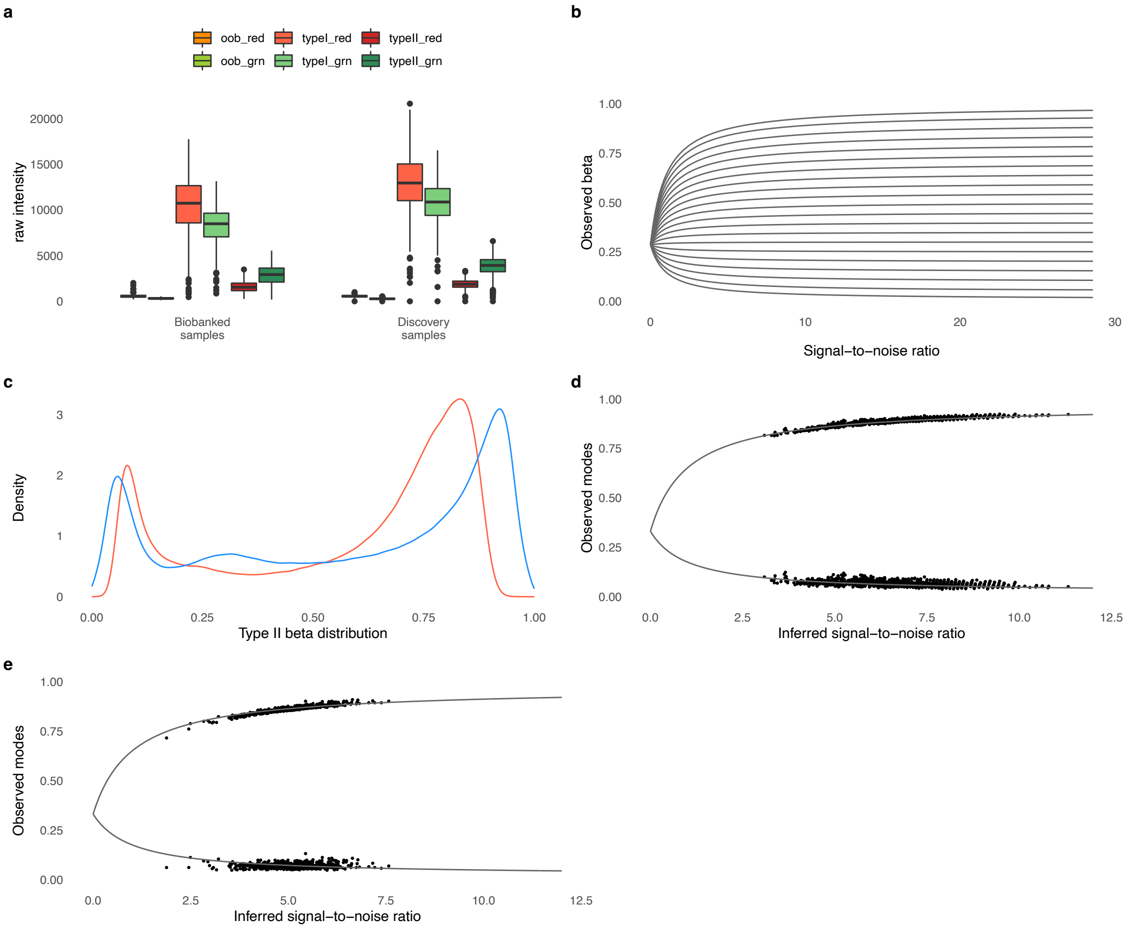

Supplementary Figure 6. **Signal-to-noise inference.** **a** Raw red and green intensities in the biobanked and discovery datasets. n=493 biobanked samples, 882 discovery samples. Box plots correspond to standard Tukey representation, with boxes indicating mean and interquartile range, and lines indicating smallest and largest values within 1.5 times of the 25th and 75th percentile, respectively. Dots indicate outlier values. **b** Numerical simulation example. A range of example beta values (represented using grey lines) become distorted at lower signal-to-noise levels. **c** Example of a high signal-to-noise ratio sample in blue (with modes close to zero and one) and a low signal-tonoise ratio sample in red (modes 'squashed' away from zero and one). The empirical densities correspond to the global type II raw beta distributions. **d** Discovery set samples. Each sample is represented by a pair of points corresponding to the methylated and unmethylated modes (based on the type II raw beta value distribution). The values along the x-axis are inferred signal-tonoise estimates for each sample. The grey lines correspond to the theoretical location of the modes as a function of signal-to-noise using equations (2) and (3) from the Supplementary Methods. **e** Biobanked samples. Each sample is represented by a pair of points corresponding to the methylated and unmethylated modes (based on the type II raw beta value distribution). The values along the x-axis are inferred signal-to-noise estimates for each sample. The grey lines correspond to the theoretical location of the modes as a function of signal-to-noise using equations (2) and (3) from the Supplementary Methods. Source data are provided as a Source Data file.

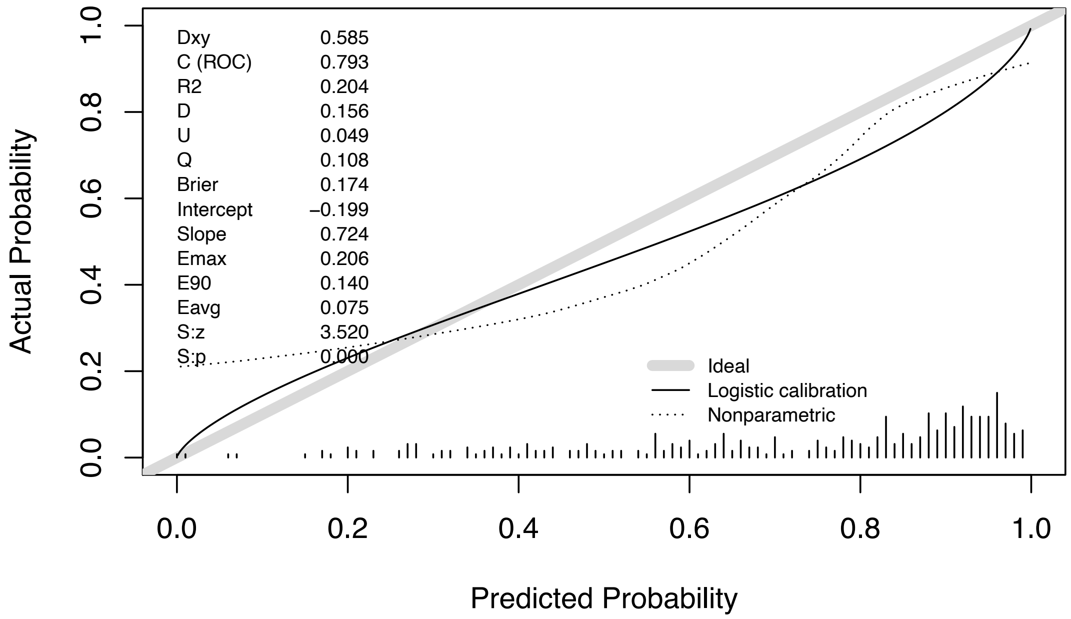

Supplementary Figure 7. **Predictive performance assessment of the WID-BC-index.** Calibration curve corresponding to the WID-BC-index in the external validation set, after recalibration based on the internal validation set. Computed using the rms R package.

Supplementary Table 1. **Epidemiological characteristics of the discovery set.** Odds ratios and p-values were calculated using median-unbiased estimation. Source data are provided as a Source Data file.

	Control (n=869)	Cancer (n=329)	OR (95% CI)	p-value
**Age (years)**				
<52	538 (62%)	162 (49%)	1.00 (reference)	-
52-64	208 (24%)	96 (29%)	1.53 (1.13, 2.06)	0.006
>64	123 (14%)	71 (22%)	1.92 (1.36, 2.69)	$<10^{-3}$
**Age at menarche (years)**				
<12	150 (17%)	67 (20%)	1.45 (1, 2.12)	0.05
12-13	437 (50%)	174 (53%)	1.3 (0.96, 1.76)	0.09
>13	277 (32%)	85 (26%)	1.00 (reference)	-
Missing or Unknown	5 (1%)	3 (1%)	-	-
**Parous**				
Yes	636 (73%)	263 (80%)	1.00 (reference)	-
No	232 (27%)	65 (20%)	0.68 (0.49, 0.92)	0.01
Missing or Unknown	1 (0%)	1 (0%)	-	-
**Age at first live child birth**				
<21	22 (3%)	10 (3%)	1.00 (reference)	-
21-27	211 (24%)	83 (25%)	0.86 (0.4, 1.98)	0.71
>27	396 (46%)	167 (51%)	0.92 (0.43, 2.09)	0.83
Missing or Unknown	240 (28%)	69 (21%)	-	-
**Menopausal status**				
Pre	509 (59%)	159 (48%)	1.00 (reference)	-
Post	360 (41%)	170 (52%)	1.51 (1.17, 1.95)	0.002
**Age at menopause (years)**				
<46	423 (49%)	105 (32%)	0.36 (0.25, 0.53)	$<10^{-6}$
46-52	260 (30%)	138 (42%)	0.78 (0.54, 1.13)	0.18
>52	101 (12%)	69 (21%)	1.00 (reference)	-
Missing or Unknown	85 (10%)	17 (5%)	-	-
**BMI (kg/m2)**				
<25	445 (51%)	201 (61%)	1.00 (reference)	-
25-30	275 (32%)	81 (25%)	0.65 (0.48, 0.88)	0.005
>30	149 (17%)	47 (14%)	0.7 (0.48, 1.01)	0.05
**Oral contraceptive use (premenopausal)**				
No	406 (80%)	147 (92%)	1.00 (reference)	-
Yes	92 (18%)	11 (7%)	0.33 (0.16, 0.62)	$<10^{-3}$
Missing or Unknown	11 (2%)	1 (1%)	-	-
**Hormone replacement therapy (postmenopausal)**				
No	306 (85%)	163 (96%)	1.00 (reference)	-
Yes	50 (14%)	7 (4%)	0.27 (0.11, 0.57)	$<10^{-3}$
Missing or Unknown	4 (1%)	0 (0%)	-	-
**Smoking**				
No	759 (87%)	290 (88%)	1.00 (reference)	-
Yes	95 (11%)	39 (12%)	1.08 (0.72, 1.59)	0.72
Missing or Unknown	15 (2%)	0 (0%)	-	-
**First degree relatives with breast cancer**				
0	770 (89%)	274 (83%)	1.00 (reference)	-
1	95 (11%)	54 (16%)	1.6 (1.11, 2.29)	0.01
2	4 (0%)	1 (0%)	0.77 (0.03, 5.62)	0.83

Supplementary Table 2. **Epidemiological characteristics of the external validation set.** Odds ratios and p-values were calculated using median-unbiased estimation. Source data are provided as a Source Data file.

	Control (n=225)	Cancer (n=113)	OR (95% CI)	p-value
**Age (years)**				
<52	91 (40%)	61 (54%)	1.00 (reference)	-
52-64	85 (38%)	27 (24%)	0.48 (0.27, 0.81)	0.006
>64	49 (22%)	25 (22%)	0.76 (0.42, 1.36)	0.36
**Age at menarche (years)**				
<12	34 (15%)	31 (27%)	1.87 (0.99, 3.54)	0.05
12-13	119 (53%)	47 (42%)	0.81 (0.48, 1.38)	0.44
>13	72 (32%)	35 (31%)	1.00 (reference)	-
**Parous**				
Yes	166 (74%)	81 (72%)	1.00 (reference)	-
No	59 (26%)	32 (28%)	1.11 (0.67, 1.84)	0.68
**Age at first live child birth**				
<21	16 (7%)	1 (1%)	1.00 (reference)	-
21-27	61 (27%)	20 (18%)	4.61 (0.84, 116.32)	0.08
>27	84 (37%)	59 (52%)	9.85 (1.92, 241.73)	0.003
Missing or Unknown	64 (28%)	33 (29%)	-	-
**Menopausal status**				
Pre	79 (35%)	57 (50%)	1.00 (reference)	-
Post	146 (65%)	56 (50%)	0.53 (0.34, 0.84)	0.007
**Age at menopause (years)**				
<46	82 (36%)	36 (32%)	1.08 (0.56, 2.13)	0.81
46-52	91 (40%)	53 (47%)	1.43 (0.77, 2.75)	0.26
>52	47 (21%)	19 (17%)	1.00 (reference)	-
Missing or Unknown	5 (2%)	5 (4%)	-	-
**BMI (kg/m2)**				
<25	117 (52%)	67 (59%)	1.00 (reference)	-
25-30	57 (25%)	29 (26%)	0.89 (0.51, 1.52)	0.67
>30	51 (23%)	17 (15%)	0.59 (0.31, 1.08)	0.09
**Oral contraceptive use (premenopausal)**				
No	28 (35%)	15 (26%)	1.00 (reference)	-
Yes	50 (63%)	42 (74%)	1.56 (0.74, 3.37)	0.25
Missing or Unknown	1 (1%)	0 (0%)	-	-
**Hormone replacement therapy (postmenopausal)**				
No	113 (77%)	46 (82%)	1.00 (reference)	-
Yes	33 (23%)	10 (18%)	0.75 (0.33, 1.61)	0.47
**Smoking**				
No	190 (84%)	100 (88%)	1.00 (reference)	-
Yes	34 (15%)	13 (12%)	0.73 (0.36, 1.42)	0.37
Missing or Unknown	1 (1%)	0 (0%)	-	-
**First degree relatives with breast cancer**				
0	193 (86%)	98 (87%)	1.00 (reference)	-
1	30 (13%)	14 (12%)	0.92 (0.45, 1.8)	0.82
2	2 (1%)	1 (1%)	1.05 (0.03, 13.08)	0.97

Supplementary Table 3. **Clinical characteristics of cancer cases in the discovery and external validation set.**

	Discovery set n=329	External validation set n=113
**Age (years)**		
<52	162 (49%)	61 (54%)
52-64	96 (29%)	27 (24%)
>64	71 (22%)	25 (22%)
**Menopausal status**		
Pre	159 (48%)	57 (50%)
Post	170 (52%)	56 (50%)
**T stage**		
T1	142 (43%)	51 (45%)
T2	163 (50%)	49 (43%)
T3/T4	22 (7%)	13 (12%)
Missing or unknown	2 (1%)	0 (0%)
**N stage**		
N0	153 (47%)	49 (43%)
>N0	167 (51%)	62 (55%)
Missing or unknown	9 (3%)	2 (2%)
**Estrogen receptor status**		
Positive	282 (86%)	94 (83%)
Negative	46 (14%)	17 (15%)
Missing or unknown	1 (0%)	2 (2%)
**Progesterone receptor status**		
Positive	247 (75%)	82 (73%)
Negative	79 (24%)	27 (24%)
Missing or unknown	3 (1%)	4 (3%)
**HER2 status**		
Positive	107 (33%)	48 (42%)
Negative	218 (66%)	64 (57%)
Missing or unknown	4 (1%)	1 (1%)
**Grade**		
I	21 (6%)	8 (7%)
II	135 (41%)	51 (45%)
III	170 (52%)	54 (48%)
Missing or Unknown	3 (1%)	0 (0%)
**Histology**		
Ductal	242 (74%)	93 (82%)
Lobular	54 (16%)	11 (10%)
Other	33 (10%)	9 (8%)

Supplementary Table 4. **Overview of epidemiological characteristics in the ovarian cancer set.** Odds ratios and p-values were calculated using median-unbiased estimation.

	Control (n=297)	Cancer (n=242)	OR (95% CI)	p-value
**Age (years)**				
<52	186 (63%)	62 (26%)	1.00 (reference)	-
52-64	71 (24%)	88 (36%)	3.7 (2.43,5.69)	$<10^{-9}$
>64	40 (13%)	92 (38%)	6.84 (4.31,11.06)	$<10^{-16}$
**Age at menarche (years)**				
<12	60 (20%)	51 (21%)	1.11 (0.69,1.8)	0.67
11-12	138 (46%)	114 (47%)	1.08 (0.73,1.6)	0.7
>13	98 (33%)	75 (31%)	1.00 (reference)	-
Missing or Unknown	1 (0%)	2 (1%)	-	-
**Parous**				
Yes	204 (69%)	187 (77%)	1.00 (reference)	-
No	92 (31%)	53 (22%)	0.63 (0.42,0.93)	0.02
Missing or Unknown	1 (0%)	2 (1%)	-	-
**Age at first live child birth**				
<21	4 (1%)	12 (5%)	1.00 (reference)	-
21-27	70 (24%)	106 (44%)	0.52 (0.14,1.58)	0.26
>27	128 (43%)	67 (28%)	0.18 (0.05,0.55)	0.002
Missing or Unknown	95 (32%)	57 (24%)	-	-
**Menopausal status**				
Pre	177 (60%)	50 (21%)	1.00 (reference)	-
Post	120 (40%)	192 (79%)	5.63 (3.84,8.37)	$<10^{-16}$
**Age at menopause (years)**				
<46	146 (49%)	54 (22%)	0.38 (0.22,0.66)	$<10^{-3}$
46-52	84 (28%)	111 (46%)	1.36 (0.79,2.32)	0.27
>52	38 (13%)	37 (15%)	1.00 (reference)	-
Missing or Unknown	29 (10%)	40 (17%)	-	-
**BMI (kg/m2)**				
<25	153 (52%)	123 (51%)	1.00 (reference)	-
25-30	94 (32%)	64 (26%)	0.85 (0.57,1.26)	0.41
>30	50 (17%)	55 (23%)	1.37 (0.87,2.15)	0.17
**Oral contraceptive use (premenopausal)**				
No	147 (83%)	43 (86%)	1.00 (reference)	-
Yes	27 (15%)	5 (10%)	0.65 (0.21,1.67)	0.39
Missing or Unknown	3 (2%)	2 (4%)	-	-
**Hormone replacement therapy (postmenopausal)**				
No	106 (88%)	186 (97%)	1.00 (reference)	-
Yes	13 (11%)	6 (3%)	0.27 (0.09,0.71)	0.007
Missing or Unknown	1 (1%)	0 (0%)	-	-
**Smoking**				
No	264 (89%)	200 (83%)	1.00 (reference)	-
Yes	31 (10%)	39 (16%)	1.66 (1,2.77)	0.05
Missing or Unknown	2 (1%)	3 (1%)	-	-
**First degree relatives with ovarian cancer**				
0	289 (97%)	228 (94%)	1.00 (reference)	-
1	8 (3%)	14 (6%)	2.2 (0.92,5.65)	0.08

Supplementary Table 5. **Overview of clinical characteristics in the ovarian cancer set.**

Value	Ovarian cancer (n=242)
**Age (years)**	
<52	62 (26%)
52-64	88 (36%)
>64	92 (38%)
**Menopausal status**	
Pre	50 (21%)
Post	192 (79%)
**T stage**	
T1	51 (21%)
T2	31 (13%)
T3	126 (52%)
T4	32 (13%)
Missing or Unknown	2 (1%)
**Grade**	
I	34 (14%)
II	14 (6%)
III	187 (77%)
Missing or Unknown	7 (3%)
**Histology**	
High grade serous	167 (69%)
Mucinous	13 (5%)
Clear cell	19 (8%)
Endometrioid	17 (7%)
Other	26 (11%)

Supplementary Table 6. **Overview of epidemiological characteristics in the endometrial cancer set.** Odds ratios and p-values were calculated using median-unbiased estimation.

	Control (n=297)	Cancer (n=217)	OR (95% CI)	p-value
**Age (years)**				
<52	186 (63%)	17 (8%)	1.00 (reference)	-
52-64	71 (24%)	64 (29%)	9.73 (5.43,18.27)	$<10^{-15}$
>64	40 (13%)	136 (63%)	36.41 (20.27,68.98)	$<10^{-inf}$
Missing or unknown	0 (0%)	0 (0%)	-	-
**Age at menarche (years)**				
<12	60 (20%)	37 (17%)	0.9 (0.54,1.51)	0.7
12-13	138 (46%)	111 (51%)	1.18 (0.79,1.76)	0.43
>13	98 (33%)	67 (31%)	1.00 (reference)	-
Missing or unknown	1 (0%)	2 (1%)	-	-
**Parous**				
Yes	204 (69%)	166 (76%)	1.00 (reference)	-
No	92 (31%)	50 (23%)	0.67 (0.45,1)	0.05
Missing or unknown	1 (0%)	1 (0%)	-	-
**Age at birth of first live child (years)**				
<21	4 (1%)	12 (6%)	1.00 (reference)	-
21-27	70 (24%)	96 (44%)	0.47 (0.12,1.43)	0.19
>27	128 (43%)	58 (27%)	0.16 (0.04,0.48)	$<10^{-3}$
Missing or unknown	95 (32%)	51 (24%)	-	-
**Menopausal status**				
Pre	177 (60%)	16 (7%)	1.00 (reference)	-
Post	120 (40%)	201 (93%)	18.27 (10.73,33.17)	$<10^{-inf}$
Missing or unknown	0 (0%)	0 (0%)	-	-
**Age at menopause (years)**				
<46	146 (49%)	29 (13%)	0.1 (0.06,0.18)	$<10^{-inf}$
46-52	84 (28%)	72 (33%)	0.44 (0.26,0.72)	0.001
>52	38 (13%)	75 (35%)	1.00 (reference)	-
Missing or unknown	29 (10%)	41 (19%)	-	-
**BMI (kg/m2)**				
<25	153 (52%)	58 (27%)	1.00 (reference)	-
25-30	94 (32%)	61 (28%)	1.71 (1.1,2.67)	0.02
>30	50 (17%)	98 (45%)	5.14 (3.27,8.16)	$<10^{-12}$
Missing or unknown	0 (0%)	0 (0%)	-	-
**Oral contraceptive use (pre menopausal only)**				
No	147 (83%)	16 (100%)	1.00 (reference)	-
Yes	27 (15%)	0 (0%)	NA	NA
Missing or unknown	3 (2%)	0 (0%)	-	-
**Hormone replacement therapy (post menopausal only)**				
No	106 (88%)	199 (99%)	1.00 (reference)	-
Yes	13 (11%)	1 (0%)	0.05 (0,0.24)	$<10^{-4}$
Missing or unknown	1 (1%)	1 (0%)	-	-
**Smoking**				
No	264 (89%)	200 (92%)	1.00 (reference)	-
Yes	31 (10%)	16 (7%)	0.68 (0.36,1.27)	0.24
Missing or unknown	2 (1%)	1 (0%)	-	-
**First degree relatives with endometrial cancer**				
0	292 (98%)	210 (97%)	1.00 (reference)	-
1+	5 (2%)	7 (3%)	1.93 (0.59,6.78)	0.27
Missing or unknown	0 (0%)	0 (0%)	-	-

Supplementary Table 7. **Overview of clinical characteristics in the endometrial cancer set.**

Value	Endometrial cancer (n=217)
**Age (years)**	
<52	17 (8%)
52-64	64 (29%)
>64	136 (63%)
Missing or unknown	0 (0%)
**Menopausal status**	
Pre	16 (7%)
Post	201 (93%)
Missing or unknown	0 (0%)
**Stage**	
IA	45 (21%)
IB	77 (35%)
II	17 (8%)
IIIA	18 (8%)
IIIB	12 (6%)
IIIC	25 (12%)
IVA	4 (2%)
IVB	11 (5%)
Missing or unknown	8 (4%)
**Grade**	
I	52 (24%)
II	57 (26%)
III	94 (43%)
Missing or unknown	14 (6%)
**Histology**	
Endometrioid	141 (65%)
Serous	39 (18%)
Other	37 (17%)
Missing or unknown	0 (0%)

Supplementary Table 8. **Overview of ENCODE samples used.**

Experiment Accession	Biosample type	Type
**thyroid gland**		
ENCSR001NCN	tissue	epithelial
ENCSR173NTZ	tissue	epithelial
ENCSR329WAK	tissue	epithelial
ENCSR406QEF	tissue	epithelial
**sigmoid colon**		
ENCSR002EIR	tissue	epithelial
ENCSR147FPX	tissue	epithelial
ENCSR773EPM	tissue	epithelial
ENCSR827WSS	tissue	epithelial
**transverse colon**		
ENCSR002LED	tissue	epithelial
ENCSR090CRZ	tissue	epithelial
ENCSR558ACF	tissue	epithelial
ENCSR580LHO	tissue	epithelial
**tibial nerve**		
ENCSR039CGW	tissue	non-epithelial
ENCSR061NRX	tissue	non-epithelial
ENCSR551DKY	tissue	non-epithelial
ENCSR729VBL	tissue	non-epithelial
**tibial artery**		
ENCSR050XGE	tissue	non-epithelial
ENCSR425TKT	tissue	non-epithelial
ENCSR646XKN	tissue	non-epithelial
ENCSR991SII	tissue	non-epithelial
**gastrocnemius medialis**		
ENCSR069UIN	tissue	non-epithelial
ENCSR193BIR	tissue	non-epithelial
ENCSR248EIV	tissue	non-epithelial
**bipolar neuron**		
ENCSR079OXD	in vitro differentiated cells	non-epithelial
**prostate gland**		
ENCSR080HYX	tissue	epithelial
ENCSR415PYP	tissue	epithelial
**stomach**		
ENCSR096DBW	tissue	epithelial
ENCSR340GPO	tissue	epithelial
ENCSR899UFG	tissue	epithelial
**esophagus squamous epithelium**		
ENCSR097SQO	tissue	epithelial
ENCSR154ELD	tissue	epithelial
ENCSR200LAH	tissue	epithelial
ENCSR312XVJ	tissue	epithelial
**upper lobe of left lung**		
ENCSR113TRL	tissue	epithelial
ENCSR426CDE	tissue	epithelial
ENCSR444YPR	tissue	epithelial
ENCSR493EGV	tissue	epithelial
**mammary epithelial cell**		

Experiment Accession	Biosample type	Type
ENCSR148KKY	primary cell	epithelial
ENCSR583ILE	primary cell	epithelial
**heart left ventricle**		
ENCSR190PQG	tissue	non-epithelial
ENCSR515ZCU	tissue	non-epithelial
**vagina**		
ENCSR190WYF	tissue	epithelial
ENCSR394PUR	tissue	epithelial
**Peyer's patch**		
ENCSR201NNA	tissue	non-epithelial
ENCSR467AVQ	tissue	non-epithelial
ENCSR719GFJ	tissue	non-epithelial
**lower leg skin**		
ENCSR203HAK	tissue	epithelial
ENCSR301SLO	tissue	epithelial
ENCSR461NFO	tissue	epithelial
ENCSR604PTS	tissue	epithelial
**adrenal gland**		
ENCSR209XGZ	tissue	non-epithelial
ENCSR371REA	tissue	non-epithelial
ENCSR399KXO	tissue	non-epithelial
**gastroesophageal sphincter**		
ENCSR215SBD	tissue	non-epithelial
ENCSR262IUB	tissue	non-epithelial
ENCSR598BUX	tissue	non-epithelial
ENCSR905RZU	tissue	non-epithelial
**kidney epithelial cell**		
ENCSR244HUE	primary cell	epithelial
**neural progenitor cell**		
ENCSR246VHI	in vitro differentiated cells	non-epithelial
**spleen**		
ENCSR276YFP	tissue	non-epithelial
ENCSR584HJL	tissue	non-epithelial
ENCSR756BTI	tissue	non-epithelial
**right atrium auricular region**		
ENCSR280LMY	tissue	non-epithelial
ENCSR517JQA	tissue	non-epithelial
**testis**		
ENCSR304AIL	tissue	epithelial
ENCSR942OLI	tissue	epithelial
**omental fat pad**		
ENCSR306JCS	tissue	non-epithelial
ENCSR662NBA	tissue	non-epithelial
ENCSR733WXF	tissue	non-epithelial
ENCSR995PGW	tissue	non-epithelial
**subcutaneous adipose tissue**		
ENCSR315CVG	in vitro differentiated cells	non-epithelial
ENCSR418YFM	tissue	non-epithelial
ENCSR733HHJ	tissue	non-epithelial
ENCSR962JMK	tissue	non-epithelial

Experiment Accession	Biosample type	Type
**skeletal muscle myoblast**		
ENCSR343SAU	primary cell	non-epithelial
ENCSR738XQU	primary cell	non-epithelial
**suprapubic skin**		
ENCSR353IUV	tissue	epithelial
ENCSR448FCV	tissue	epithelial
ENCSR575WOH	tissue	epithelial
ENCSR792ATG	tissue	epithelial
**breast epithelium**		
ENCSR392LYN	tissue	epithelial
ENCSR393CCK	in vitro differentiated cells	epithelial
ENCSR846DDC	tissue	epithelial
**smooth muscle cell**		
ENCSR420WUN	in vitro differentiated cells	non-epithelial
**epithelial cell of alveolus of lung**		
ENCSR422EPB	primary cell	epithelial
**cardiac muscle cell**		
ENCSR449VMS	tissue	non-epithelial
**myotube**		
ENCSR468IFF	in vitro differentiated cells	non-epithelial
ENCSR809OPY	primary cell	non-epithelial
**esophagus muscularis mucosa**		
ENCSR472PKR	tissue	epithelial
ENCSR701SVQ	tissue	epithelial
ENCSR822VTU	tissue	epithelial
ENCSR871SFO	tissue	epithelial
**ascending aorta**		
ENCSR486SMB	tissue	non-epithelial
ENCSR731SPT	tissue	non-epithelial
**ovary**		
ENCSR511SNB	tissue	epithelial
ENCSR744AJV	tissue	epithelial
**non-pigmented ciliary epithelial cell**		
ENCSR528NFI	primary cell	epithelial
**coronary artery**		
ENCSR582BMR	tissue	non-epithelial
ENCSR688OHW	tissue	non-epithelial
**body of pancreas**		
ENCSR597BUD	tissue	epithelial
ENCSR705PDD	tissue	epithelial
ENCSR922EBK	tissue	epithelial
ENCSR940ZHS	tissue	epithelial
**epithelial cell of proximal tubule**		
ENCSR675IVH	primary cell	epithelial
**iris pigment epithelial cell**		
ENCSR754ANZ	primary cell	epithelial
**uterus**		
ENCSR803DDS	tissue	epithelial
ENCSR889TZA	tissue	epithelial
**astrocyte**		

Experiment Accession	Biosample type	Type
ENCSR847BAX	primary cell	non-epithelial
**retinal pigment epithelial cell**		
ENCSR899LHQ	primary cell	epithelial
**right lobe of liver**		
ENCSR937LYZ	tissue	epithelial
**hepatocyte**		
ENCSR955LKF	primary cell	epithelial
**renal cortical epithelial cell**		
ENCSR963NNG	primary cell	epithelial
**choroid plexus epithelial cell**		
ENCSR976HYM	tissue	non-epithelial

Supplementary Table 9. **Overview of breast cancer cases and control cases collected in different countries (discovery set).**

Country	Breast cancer (n=329)	Control (n=869)	Total (n=1198)
Czech Republic	35	222	257
Germany	47	2	49
Italy	228	61	289
Norway	0	163	163
UK	19	421	440

Supplementary Table 10. **Overview of normal, *BRCA* carrier, and normal adjacent (from women with triple negative breast cancer) tissue samples.**

Type	Mean age (years)	Total (n=42)
Normal (cosmetic surgery)	31	14
BRCA1 (risk reducing surgery)	36	9
BRCA2 (risk reducing surgery)	36	5
Normal adjacent	43	14

Supplementary References

1. Perskvist N, Bjorklund C, Dillner J. A complex intervention for workflow enhancement at the Swedish cervical cytology biobank. *Biopreserv Biobank* 2014; **12**(1): 69-73.
2. Perskvist N, Norman I, Eklund C, Litton JE, Dillner J. The Swedish cervical cytology biobank: sample handling and storage process. *Biopreserv Biobank* 2013; **11**(1): 19-24.
3. Zhou W, Laird PW, Shen H. Comprehensive characterization, annotation and innovative use of Infinium DNA methylation BeadChip probes. *Nucleic Acids Res* 2017; **45**(4): e22.
4. Van Calster B, McLernon DJ, van Smeden M, et al. Calibration: the Achilles heel of predictive analytics. *BMC Med* 2019; **17**(1): 230.
5. Mohammed H, Russell IA, Stark R, et al. Progesterone receptor modulates ERalpha action in breast cancer. *Nature* 2015; **523**(7560): 313-7.
6. Yin P, Roqueiro D, Huang L, et al. Genome-wide progesterone receptor binding: cell type-specific and shared mechanisms in T47D breast cancer cells and primary leiomyoma cells. *PLoS One* 2012; **7**(1): e29021.
7. Purcell S, Neale B, Todd-Brown K, et al. PLINK: a tool set for whole-genome association and population-based linkage analyses. *Am J Hum Genet* 2007; **81**(3): 559-75.
8. Manichaikul A, Mychaleckyj JC, Rich SS, Daly K, Sale M, Chen WM. Robust relationship inference in genome-wide association studies. *Bioinformatics* 2010; **26**(22): 2867-73.
9. Das S, Forer L, Schonherr S, et al. Next-generation genotype imputation service and methods. *Nat Genet* 2016; **48**(10): 1284-7.
10. Mavaddat N, Michailidou K, Dennis J, et al. Polygenic Risk Scores for Prediction of Breast Cancer and Breast Cancer Subtypes. *Am J Hum Genet* 2019; **104**(1): 21-34.
11. Michailidou K, Lindstrom S, Dennis J, et al. Association analysis identifies 65 new breast cancer risk loci. *Nature* 2017; **551**(7678): 92-4.
12. Hosseini H, Obradovic MMS, Hoffmann M, et al. Early dissemination seeds metastasis in breast cancer. *Nature* 2016; **540**(7634): 552-8.
13. Chlebowski RT, Anderson GL, Aragaki AK, et al. Association of Menopausal Hormone Therapy With Breast Cancer Incidence and Mortality During Long-term Follow-up of the Women's Health Initiative Randomized Clinical Trials. *JAMA* 2020; **324**(4): 369-80.
14. Lacasa D, Le Liepvre X, Ferre P, Dugail I. Progesterone stimulates adipocyte determination and differentiation 1/sterol regulatory element-binding protein 1c gene expression. potential mechanism for the lipogenic effect of progesterone in adipose tissue. *J Biol Chem* 2001; **276**(15): 11512-6.
15. Esslimani-Sahla M, Thezenas S, Simony-Lafontaine J, et al. Increased expression of fatty acid synthase and progesterone receptor in early steps of human mammary carcinogenesis. *Int J Cancer* 2007; **120**(2): 224-9.
16. Argov-Argaman N, Raz C, Roth Z. Progesterone Regulation of Milk Fat Globule Size Is VLDL Dependent. *Front Endocrinol (Lausanne)* 2020; **11**: 596.
17. Poole AJ, Li Y, Kim Y, Lin SC, Lee WH, Lee EY. Prevention of Brca1-mediated mammary tumorigenesis in mice by a progesterone antagonist. *Science* 2006; **314**(5804): 1467-70.

18. Aryee MJ, Jaffe AE, Corrada-Bravo H, et al. Minfi: a flexible and comprehensive Bioconductor package for the analysis of Infinium DNA methylation microarrays. *Bioinformatics* 2014; **30**(10): 1363-9.
19. Morris TJ, Butcher LM, Feber A, et al. ChAMP: 450k Chip Analysis Methylation Pipeline. *Bioinformatics* 2014; **30**(3): 428-30.
20. Friedman J, Hastie T, Tibshirani R. Regularization Paths for Generalized Linear Models via Coordinate Descent. *J Stat Softw* 2010; **33**(1): 1-22.
21. Sergushichev AA. An algorithm for fast preranked gene set enrichment analysis using cumulative statistic calculation. 2016: 060012.
22. Price AL, Patterson NJ, Plenge RM, Weinblatt ME, Shadick NA, Reich D. Principal components analysis corrects for stratification in genome-wide association studies. *Nat Genet* 2006; **38**(8): 904-9.
23. International HapMap C. The International HapMap Project. *Nature* 2003; **426**(6968): 789-96.
