# Supplementary material for: The WID-BC-index identifies women with primary poor prognostic breast cancer based on DNA methylation in cervical samples
Source: Nat Commun. 2022 Feb 1;13:449. doi: 10.1038/s41467-021-27918-w (PMC8807602; doi:10.1038/s41467-021-27918-w)
Supplement: Supplementary file 3 — Reporting Summary [file 41467_2021_27918_MOESM3_ESM.pdf]

nature portfolio

Corresponding author(s): Martin Widschwendter
Last updated by author(s): Nov 9, 2021

Reporting Summary

Nature Portfolio wishes to improve the reproducibility of the work that we publish. This form provides structure for consistency and transparency in reporting. For further information on Nature Portfolio policies, see our Editorial Policies and the Editorial Policy Checklist.

Statistics

For all statistical analyses, confirm that the following items are present in the figure legend, table legend, main text, or Methods section.

n/a	Confirmed	
☐	☒	The exact sample size (*n*) for each experimental group/condition, given as a discrete number and unit of measurement
☐	☒	A statement on whether measurements were taken from distinct samples or whether the same sample was measured repeatedly
☐	☒	The statistical test(s) used AND whether they are one- or two-sided<br>*Only common tests should be described solely by name; describe more complex techniques in the Methods section.*
☐	☒	A description of all covariates tested
☐	☒	A description of any assumptions or corrections, such as tests of normality and adjustment for multiple comparisons
☐	☒	A full description of the statistical parameters including central tendency (e.g. means) or other basic estimates (e.g. regression coefficient) AND variation (e.g. standard deviation) or associated estimates of uncertainty (e.g. confidence intervals)
☐	☒	For null hypothesis testing, the test statistic (e.g. *F*, *t*, *r*) with confidence intervals, effect sizes, degrees of freedom and *P* value noted<br>*Give P values as exact values whenever suitable.*
☒	☐	For Bayesian analysis, information on the choice of priors and Markov chain Monte Carlo settings
☒	☐	For hierarchical and complex designs, identification of the appropriate level for tests and full reporting of outcomes
☐	☒	Estimates of effect sizes (e.g. Cohen's *d*, Pearson's *r*), indicating how they were calculated

Our web collection on statistics for biologists contains articles on many of the points above.

Software and code

Policy information about availability of computer code

Data collection: No proprietary software was used for data collection; questionnaires were acquired using the Qualtrics application on dedicated iPads.

Data analysis: The following R packages were used:
- Minfi, version 1.36.0 [Aryee, Martin J., et al. "Minfi: a flexible and comprehensive Bioconductor package for the analysis of Infinium DNA methylation microarrays." Bioinformatics 30.10 (2014): 1363-1369].
- impute, version 1.62.0 [Hastie T, Tibshirani R, Narasimhan B, Chu G (2021). impute: impute: Imputation for microarray data.]
- ChAMP, version 2.18.3 [Jerome Friedman, Trevor Hastie, Robert Tibshirani. “Regularization Paths for Generalized Linear Models via Coordinate Descent.” Journal of Statistical Software, 33(1) (2010), 1-22.].
- glmnet, version 2.0-18 [Sergushichev A. “An algorithm for fast preranked gene set enrichment analysis using cumulative statistic calculation.” bioRxiv. doi: 10.1101/060012 (2016)].
- EpiDISH, version 2.6.1 (downloaded from https://github.com/sjczheng/EpiDISH).
- pROC, version 1.18.0 [downloaded from CRAN; Xavier Robin, Natacha Turck, Alexandre Hainard, et al. (2011) “pROC: an open-source package for R and S+ to analyze and compare ROC curves”. BMC Bioinformatics, 7, 77. DOI: 10.1186/1471-2105-12-77]
- iterativeBMA, version 1.42.0 [accessed via Bioconductor; Yeung KY, Washington Uo, Seattle, WA, Raftery wcfA, Painter I (2021). iterativeBMA: The Iterative Bayesian Model Averaging (BMA) algorithm. http://faculty.washington.edu/kayee/research.html.]
- rms, version 6.1-1. [accessed via CRAN; Harrell, Frank; https://cran.r-project.org/web/packages/rms/index.html]
- GCTA version 1.26.0 [Yang J, Lee SH, Goddard ME, Visscher PM (2011). GCTA: A Tool for Genome-wide Complex Trait aAnalysis. American Journal for Human Genetics. doi: 10.1016/j.ajhg.2010.11.011]
- PLINK version 1.9 [https://zzz.bwh.harvard.edu/plink/]
- TCGAbiolinks, version 2.16.4 [Colaprico A, Silva TC, Olsen C, Garofano L, Cava C, Garolini D, Sabedot T, Malta TM, Pagnotta SM, Castiglioni I, Ceccarelli M, Bontempi G, Noushmehr H (2015). “ TCGAbiolinks: An R/Bioconductor package for integrative analysis of TCGA data.” Nucleic Acids Research. doi: 10.1093/nar/gkv1507, http://doi.org/10.1093/nar/gkv1507.]
- GEOquery, version 2.58.0 [Davis S, Meltzer P (2007). “GEOquery: a bridge between the Gene Expression Omnibus (GEO) and BioConductor.”

Bioinformatics, 14, 1846–1847.]
- KING [Kinship-based INference for Gwas, https://www.kingrelatedness.com/]
- Michigan Imputation Server [https://imputationserver.sph.umich.edu/index.html]
- WID.BC package, version 1.0 [Herzog., C., Barrett, J., Widschwendter, M. The WID-BC-index identifies women with primary poor prognostic breast cancer based on DNA methylation in cervical samples. WID.BC. doi.org/10.5281/zenodo.5651989 (2021)]

For manuscripts utilizing custom algorithms or software that are central to the research but not yet described in published literature, software must be made available to editors and reviewers. We strongly encourage code deposition in a community repository (e.g. GitHub). See the Nature Portfolio guidelines for submitting code & software for further information.

Data

Policy information about availability of data

All manuscripts must include a data availability statement. This statement should provide the following information, where applicable:

- Accession codes, unique identifiers, or web links for publicly available datasets
- A description of any restrictions on data availability
- For clinical datasets or third party data, please ensure that the statement adheres to our policy

Previously published data were accessed from ENCODE (accession codes listed in Supplementary Table 3), GEO (GSE40724 [https://www.ncbi.nlm.nih.gov/geo/query/acc.cgi?acc=GSE40724]) and GSE68355 [https://www.ncbi.nlm.nih.gov/geo/query/acc.cgi?acc=GSE68355]), and TCGA (TCGA-BRCA project [https://portal.gdc.cancer.gov/projects/TCGA-BRCA]). Raw DNAme and SNP data generated in this study have been deposited in the European Genome-phenome Archive (EGA) database under the study accession codes EGAS00001005055 [https://ega-archive.org/studies/EGAS00001005055] (breast cancer cervical and buccal methylation methylation and PRS SNP data), EGAS00001005070 [https://ega-archive.org/studies/EGAS00001005070] (breast tissue methylation), EGAS00001005045 [https://ega-archive.org/studies/EGAS00001005045] (ovarian cancer cervical methylation), EGAS00001005033 [https://ega-archive.org/studies/EGAS00001005033] (endometrial cancer cervical methylation data), and EGAS00001005626 [https://ega-archive.org/studies/EGAS00001005626] (matched methylation data from cervical, buccal, and blood samples from controls and BRCA1/2 mutation carriers). The raw data are available under restricted access due to patient confidentiality and privacy laws. Access can be obtained by formal application to the relevant Data Access Committee via EGA and signing of a Data Access Agreement. Source data for this paper are provided under https://github.com/chiaraherzog/WID-BC-source-data.

Field-specific reporting

Please select the one below that is the best fit for your research. If you are not sure, read the appropriate sections before making your selection.

☒ Life sciences ☐ Behavioural & social sciences ☐ Ecological, evolutionary & environmental sciences

For a reference copy of the document with all sections, see nature.com/documents/nr-reporting-summary-flat.pdf

Life sciences study design

All studies must disclose on these points even when the disclosure is negative.

Sample size	Justification for the proposed sample size of the discovery set was based on the outcome of previous prospective EWAS studies performed by us, as well as independent EWAS power calculations (Rakyan VK, Down TA, Balding DJ, Beck S. Epigenome-wide association studies for common human diseases. Nat Rev Genet. 2011;12(8):529-41) both converging on similar estimates.
Data exclusions	No data were excluded.
Replication	This was a study based on samples collected from individuals with breast cancer or controls. Samples were taken at a single timepoint from individuals with breast cancer or controls.
Randomization	No treatment was carried out. Groups were assigned to presence ("breast cancer" individuals) or absence ("control" individuals) of breast cancer.
Blinding	No treatment was carried out as part of this study, only sample collection. Investigators collecting sample were health practitioners in touch with the patients and as such were not blinded to patient status, but blinding was not required in this context.

Reporting for specific materials, systems and methods

We require information from authors about some types of materials, experimental systems and methods used in many studies. Here, indicate whether each material, system or method listed is relevant to your study. If you are not sure if a list item applies to your research, read the appropriate section before selecting a response.

Materials & experimental systems

n/a	Involved in the study	
☒	☐	Antibodies
☒	☐	Eukaryotic cell lines
☒	☐	Palaeontology and archaeology
☒	☐	Animals and other organisms
☐	☒	Human research participants
☒	☐	Clinical data
☒	☐	Dual use research of concern

Methods

n/a	Involved in the study	
☒	☐	ChIP-seq
☒	☐	Flow cytometry
☒	☐	MRI-based neuroimaging

Human research participants

Policy information about studies involving human research participants

Population characteristics	All participants were female. Detailed description of characteristics of the various study groups is provided in the manuscript. Biological samples were given an anonymous Participant ID Number which was assigned to the person's name in a securely stored link file. Women with a current diagnosis of a primary breast cancer and recruited prior to receiving any systemic treatment (chemotherapy, etc.) or surgery were eligible as brast cancer cases. Controls were initially matched (to all cancer cases in FORECEE) one-to-one with cases based on menopausal status, age (5 year age ranges where possible), and recruitment centre/country. However, due to an imbalance in recruitment of cases and controls at some centres, a number of cases were matched on age and menopausal status alone. Cancer histological data was collected post-recruitment either by clinicians directly involved in the diagnosis/treatment of the cancer cases or by a nominated data manager with access to the in-house hospital systems.
Recruitment	The study was conducted as part of a multi-centre study involving several recruitment sites in 5 European countries (i.e. the UK, Czech Republic, Italy, Norway and Germany). Participants were aged >18 years. Prior to taking part, each prospective study volunteer was given a Participant Information Sheet as well as a Consent Form and the rationale for the study was explained. Additional resources, including an explanatory video and further online resources, were also made available. Women diagnosed with breast cancer (case) or a non-malignant benign gynaecological condition (control) were approached during outpatient hospital clinics, while women recruited as healthy volunteers from the general population (control) were approached via outreach campaigns, public engagement, and as part of cervical screening programmes. After signing an informed consent, participants completed an epidemiological questionnaire as well as a feedback form after their participation.
Ethics oversight	The case-control study presented in this manuscript is a sub-study of the FORECEE (4C) programme which was conducted as part of a multi-centre study involving several recruitment sites in 5 European countries (i.e. the UK, Czech Republic, Italy, Norway, Germany) (Supplementary Table 9) and has ethical approval from UK Health Research Authority (REC 14/LO/1633) and all contributing centres, including the NRES Committee London (UK), Ethics Committee of the General University Hospital, Prague (Czech Republic), Comitato Etico degli IRCSS Instituto Europeo di Oncologia e Centro Cardiologico Monzino (Itality), Regionale Komiteer for Medisinsk og Helsefaglig Forskningsetikk (Norway), and Ethikkommission bei der LMU München (Germany).<br>Ethical approval for the population-based cohort samples was granted by Ethical approval was granted by the Karolinska Ethical Committee Dnr 2014/1242-31/4 and Dnr 2016/304-32.

Note that full information on the approval of the study protocol must also be provided in the manuscript.
